# SoupX removes ambient RNA contamination from droplet-based single-cell RNA sequencing data

**DOI:** 10.1093/gigascience/giaa151

**Published:** 2020-12-26

**Authors:** Matthew D Young, Sam Behjati

**Affiliations:** Wellcome Trust Sanger Institute, Cellular Genetics, Wellcome Genome Campus, Hinxton, CB10 1SA, UK; Wellcome Trust Sanger Institute, Cellular Genetics, Wellcome Genome Campus, Hinxton, CB10 1SA, UK; Cambridge University Hospitals NHS Foundation Trust, Hills Road, Cambridge, CB2 0QQ, UK; University of Cambridge, Department of Paediatrics, Cambridge Biomedical Campus, Cambridge, CB2 0QQ, UK

**Keywords:** scRNA-seq, decontamination, pre-processing

## Abstract

**Background:**

Droplet-based single-cell RNA sequence analyses assume that all acquired RNAs are endogenous to cells. However, any cell-free RNAs contained within the input solution are also captured by these assays. This sequencing of cell-free RNA constitutes a background contamination that confounds the biological interpretation of single-cell transcriptomic data.

**Results:**

We demonstrate that contamination from this "soup" of cell-free RNAs is ubiquitous, with experiment-specific variations in composition and magnitude. We present a method, SoupX, for quantifying the extent of the contamination and estimating "background-corrected" cell expression profiles that seamlessly integrate with existing downstream analysis tools. Applying this method to several datasets using multiple droplet sequencing technologies, we demonstrate that its application improves biological interpretation of otherwise misleading data, as well as improving quality control metrics.

**Conclusions:**

We present SoupX, a tool for removing ambient RNA contamination from droplet-based single-cell RNA sequencing experiments. This tool has broad applicability, and its application can improve the biological utility of existing and future datasets.

## Introduction

Droplet-based single-cell RNA sequencing (scRNA-seq) has enabled quantification of the transcriptomes of hundreds of thousands of cells in single experiments [1,2]. This technology underpins recent advances in understanding normal and pathological cell behaviour [3–8]. Moreover, large-scale efforts to create a ”Human Cell Atlas" critically depend on the accuracy and cellular specificity of the transcriptional readout produced by droplet-based scRNA-seq [9,10].

A core assumption of droplet-based scRNA-seq is that each droplet, within which molecular tagging and reverse transcription take place, contains messenger RNA (mRNA) from a single cell. Violations of this assumption, which may distort the interpretation of scRNA-seq data, are common in practice. Clear examples include droplets that contain multiple cells (doublets), and empty droplets. Attempts to detect and remove doublets are an active area of research [11–13].

Another phenomenon that violates this assumption is the sequencing of cell-free RNA from the input solution, admixed with a cell in its enclosing droplet. It is recognized that these contaminating non-endogenous RNAs are present even within datasets of the highest quality [2]. Here, we show that this "soup" of cell-free mRNAs is ubiquitous and non-negligible in magnitude. Because the character and extent of ambient mRNA contamination varies by experiment, with increased contamination in necrotic or complex samples, ambient mRNAs may significantly confound the biological interpretation of scRNA-seq data. We present SoupX, a method for quantifying the extent of ambient mRNA contamination whilst purifying the true, cell-specific signal from the observed mixture of cellular and exogenous mRNAs.

In this article we begin by briefly describing the SoupX method. Following this we consider a range of datasets, summarized in Supplementary Table S1. We first investigate 2 “species mixing” datasets run on the Chromium 10X [2] and DropSeq [14] platforms, which allow us to directly identify contaminating mRNAs and test our method’s accuracy. We then demonstrate how SoupX can be applied in practice using a dataset of peripheral blood mononuclear cells (PBMCs) [2]. We further explore the biological benefits of SoupX using a complex "kidney tumour" dataset, which consists of 12 kidney tumour biopsies [15]. As a final test, we apply our method to human fetal liver data [16]. We conclude with some general remarks about ambient RNA contamination, other tools to correct for its effect, and the consequences of failing to account for the presence of ambient RNAs.

## The SoupX Method

Droplet-based scRNA-seq methods produce counts of unique molecular identifiers (UMIs) for genes in thousands of cells. The aim of an scRNA-seq experiment is to infer the number of molecules present for each type of gene within each cell from these data. However, the observed counts arise from a mixture of mRNAs produced by the captured cell and those present due to background contamination. SoupX aims to remove the contribution of the cell-free mRNA molecules from each cell and recover the true molecular abundance of each gene in each cell.

**Figure 1:**
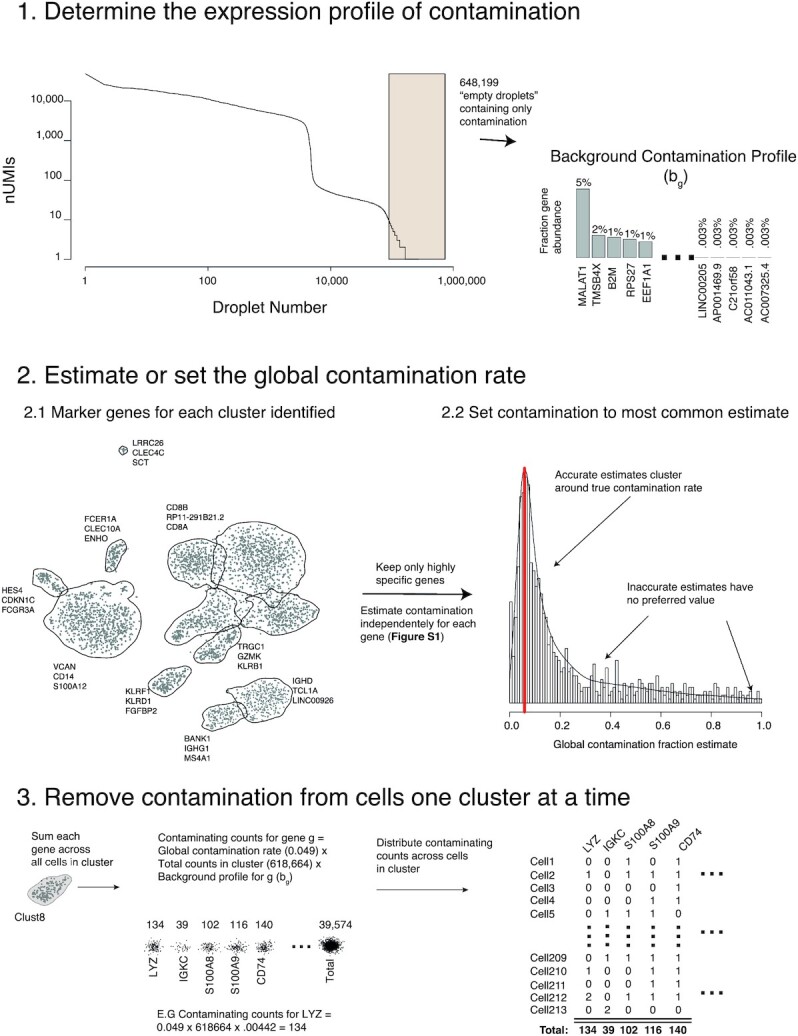
A visual summary of the SoupX method, using data from the PBMC dataset.

The algorithm consists of the following 3 steps (summarized in Fig. 1):

1. Estimate the ambient mRNA expression profile from empty droplets.
2. Estimate (or manually set) the contamination fraction, the fraction of UMIs originating from the background, in each cell.
3. Correct the expression of each cell using the ambient mRNA expression profile and estimated contamination.

SoupX produces a modified table of counts, which can be used in place of the original count matrix in any downstream analysis tool.

To estimate the background expression profile we consider all droplets with <*N*_emp_ UMIs, which we assume unambiguously do not contain cells. The fraction of background expression from gene *g*, *b_g_*, is then given by,

(1) \begin{equation*} b_{g} = \frac{\sum _d n_{g,d}}{\sum _d \sum _g n_{g,d}}, \end{equation*}

where *n_g, d_* is the number of counts for gene *g* in droplet *d* and the sum over *d* is taken over all droplets with <*N*_emp_ UMIs (Fig. 1). The species-mixing experiment allows us to compare how accurately *b_g_* recapitulates the true background expression found within each cell, revealing that any value of *N*_emp_ < 100 produces a good correlation, with the best correlation given when *N*_emp_ < 10 (Supplementary Fig. S2).

The most challenging part of using SoupX is estimating or specifying the number of UMIs in each cell that are contributed by background contamination. In general, the observed number of UMIs for gene *g* in cell *c* is given by

(2) \begin{equation*} n_{g,c} = m_{g,c} + o_{g,c}, \end{equation*}

where *m_g, c_* are the cell endogenous counts and *o_g, c_* are the counts from the background. We assume that the relative abundance of genes that make up the background does not differ between cells, which allows us to write,

(3) \begin{equation*} o_{g,c} = N_c \rho _c b_g, \end{equation*}

where *N_c_* = ∑_*g*_*n_g, c_*, and ρ_*c*_ is the background contamination fraction. In general *m_g, c_* is unknown and what we are aiming to measure. To proceed, we assume that there is a combination of genes and cells for which *m_g, c_* = 0 exists. The genes for which *m_g, c_* = 0 for a given cell are those genes that are strong negative markers of the cell type *c*. For example, the gene *HBB* is a strong positive marker for erythroid cells (red blood cells) but should not be expressed in any other cell type. So for any cell *c* that is not an erythroid cell, *HBB* will not be expressed (i.e., $m_{HBB,\mathrm{not\ Erythroid}} = 0$).

Given a set of genes/cells for which we can assume that there is no cell endogenous expression (i.e., *m_g, c_* = 0) we calculate the cell-specific contamination fraction,

(4) \begin{equation*} \rho _c = \frac{\sum _g n_{g,c}}{N_c \sum _g b_g}, \end{equation*}

where the sum is taken across all genes in cell *c* for which it is assumed *m_g, c_* = 0. SoupX optionally uses clustering information to refine the set of cells for which it can be assumed that *m_g, c_* = 0. If it can be shown for any cell *c* in cluster *P* that *m_g, c_* > 0, then it is assumed that *m_g, c_* > 0 for all *c* ∈ *P* (see Supplementary Fig. S1).

If known from prior biological knowledge, the set of genes/cells for which it can be assumed that *m_g, c_* = 0can be provided as input to SoupX. Where this is not known in advance, we provide an automated alternative to estimate the contamination fraction (see Supplementary Fig. S1). The automated approach first identifies markers of each cluster of cells in the data. For each strong marker, it is assumed that *m_g, c_* = 0 for all cells in clusters where the gene is not a marker and the contamination fraction is estimated (Supplementary Fig. S1). Performing this estimation across all strong marker genes provides a set of estimates of the contamination fraction. To obtain a final value, it is assumed that inaccurate estimates will have no preferred value while true estimates will cluster around the true value. The most common value is taken as the final estimate of the contamination fraction (see Fig. 1, Step 2.2).

Having determined the contamination fraction ρ_*c*_ and the background expression profile *b_g_*, the cell endogenous counts are intuitively given by

(5) \begin{equation*} m_{g,c} = n_{g,c} - N_c \rho _c b_g, \end{equation*}

where *n_g, c_* are the observed counts, *N_c_* = ∑_*g*_*n_g, c_*, and *b_g_* and ρ_*c*_ are calculated as described above.

Although the intuition of Equation 5 is correct, in practice *m_g, c_* is estimated by maximizing a multinomial likelihood as described in the Supplementary Methods. This procedure is further enhanced when cluster assignments are given, by performing the correction on counts aggregated at the cluster level, then distributing the corrected counts between cells in the cluster in proportion to their size (see Fig. 1). This additional step helps overcome the sparsity of scRNA-seq data, which would otherwise make it impossible to distinguish a single count due to contamination from a single count due to endogenous expression in many circumstances.

The estimated value of *m_g, c_* can then be used in place of *n_g, c_* in any downstream analysis.

## Properties of Ambient RNA

**Figure 2:**
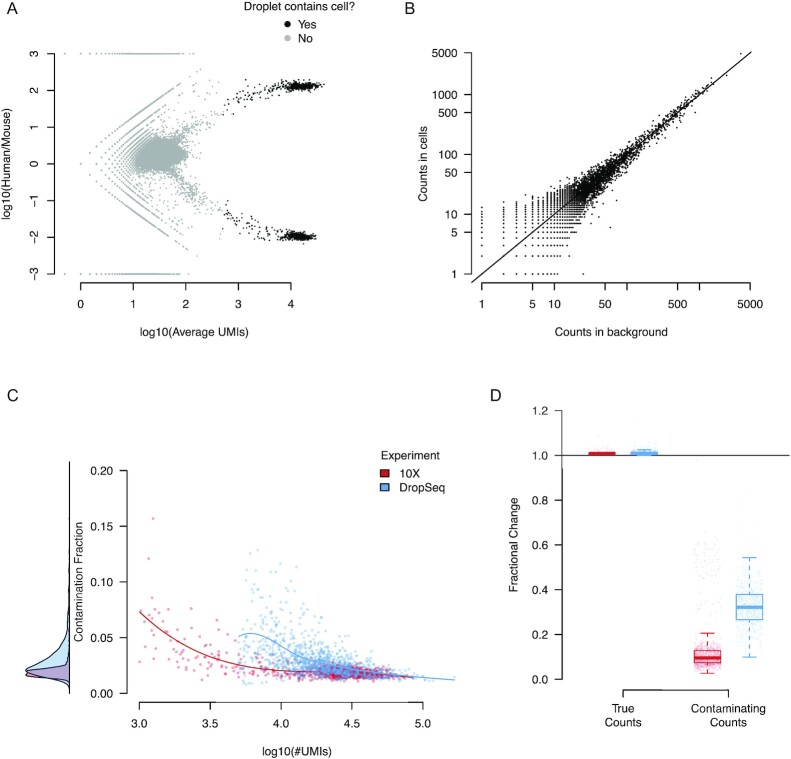
The properties of the cell-free mRNA soup as determined using species-mixing datasets. **A**, The log_10_ ratio of the number of UMIs mapping to human and mouse mRNAs for each droplet in the species-mixing dataset (10X). Droplets determined to contain cells by cellranger are marked in black. **B**, The correlation of the counts in the background compared to counts averaged across cells for each gene. Counts have been subsampled so that the total number of counts in the background and averaged cell population are the same. **C**, The estimated contamination fraction as a function of number of UMIs in each droplet in individual cells in the species-mixing dataset. Red and blue dots represent cells from the 10X/DropSeq experiments, respectively. The distribution on the left shows the marginal distribution across all cells. **D**, The fractional change in contaminating and genuine expression levels after applying SoupX for the 2 technologies. The distribution across cells is summarized by box plots, where the central line is the median, box boundaries are the first and third quartiles, and the whiskers extend to 1.5 times the interquartile range.

We next investigate the properties of ambient RNA contamination in data where ground truth is available, the “species-mixing” experiments combining mouse and human cell lines using 10X [2] and Drop-Seq technologies [14]. Figure 2A shows the relative abundance of human and mouse mRNAs in each droplet in the 10X data. Droplets containing human (top right) and mouse (bottom right) cells show that ∼1% of observed transcripts are cross-species contamination. This rate of cross-species contamination provides a lower bound on the total rate of ambient mRNA contamination because there will also be an additional contribution due to contaminating mRNAs from the same species (we later show that the true contamination rate is $\sim 2\%$). A similar effect is seen in the Drop-Seq–based species-mixing data (Supplementary Fig. S3). These observations demonstrate that cell-free mRNA contamination is present even in highly controlled experiments.

To investigate the composition of cell-free mRNAs, we compared the aggregate expression profile of all droplets containing cells to all droplets with ≤10 UMIs, which we assumed to contain only ambient mRNAs. These 2 profiles were highly correlated in the 10X species experiment (Fig. 2B) with a high correlation found in all other datasets considered (Pearson correlation 0.71–0.96, median 0.86; Supplementary Table S2). The strength of the correlation implies that cell-free contamination represents an approximately uniform sampling of the cells in the sequencing batch (i.e., channel).

Next we estimated the contamination fraction, the fraction of expression derived from the cell-free mRNA background in each cell. In each cell we identify a set of genes that must have originated from the ambient mRNA: human transcripts in mouse cells and vice versa. For these genes/cells it is assumed that *m_g, c_* = 0 and the contamination fraction is calculated using Equation 4. Figure 2C reveals that there is little variation in the contamination fraction within a channel, in both the 10X and DropSeq data.

In most experiments there is less power to determine cell-specific contamination fractions and so SoupX assumes a constant contamination fraction within a channel. When clustering information is provided, the redistribution of counts from cluster level to individual cells automatically removes more counts from contaminated cells, even when only a global estimate of the contamination is given (Supplementary Fig. S4; Supplementary Methods). Where a cell-specific expression estimate is needed, SoupX uses a hierarchical Bayes method to share information between cells (Supplementary Methods).

It may be hypothesized that the absolute number of contaminating mRNA molecules is the quantity that is approximately constant and that the contamination should vary with the number of mRNA molecules contributed by the captured cell. That is, that contamination fraction should vary as a function of cellular mRNA contribution, with the number of detected UMIs being a proxy for this. Consistent with this, Fig. 2C shows that the greatest contamination occurs in droplets with the fewest UMIs. However, the contamination fraction is still approximately constant across most of the UMI range. This is likely a consequence of the fact that the capture efficiency of molecules in droplet-based experiments varies by as much as an order of magnitude [17]. Thus variation due to capture efficiency is likely to swamp variation due to “cell size” in most experiments, making constant contamination fraction a reasonable approximation.

To test the accuracy of SoupX in removing contaminating counts while retaining those due to endogenous expression we compared the fraction of expression from cross-species and within-species genes before and after SoupX contamination correction. This analysis revealed (Fig. 2D) that mouse expression in human cells (and vice versa) was decreased by a factor of ≥2 and usually an order of magnitude by the SoupX contamination removal in both 10X and DropSeq experiments. By contrast the fraction of expression derived from genes corresponding to the correct species was effectively unchanged for all cells.

## Application of SoupX to PBMC Data

**Figure 3:**
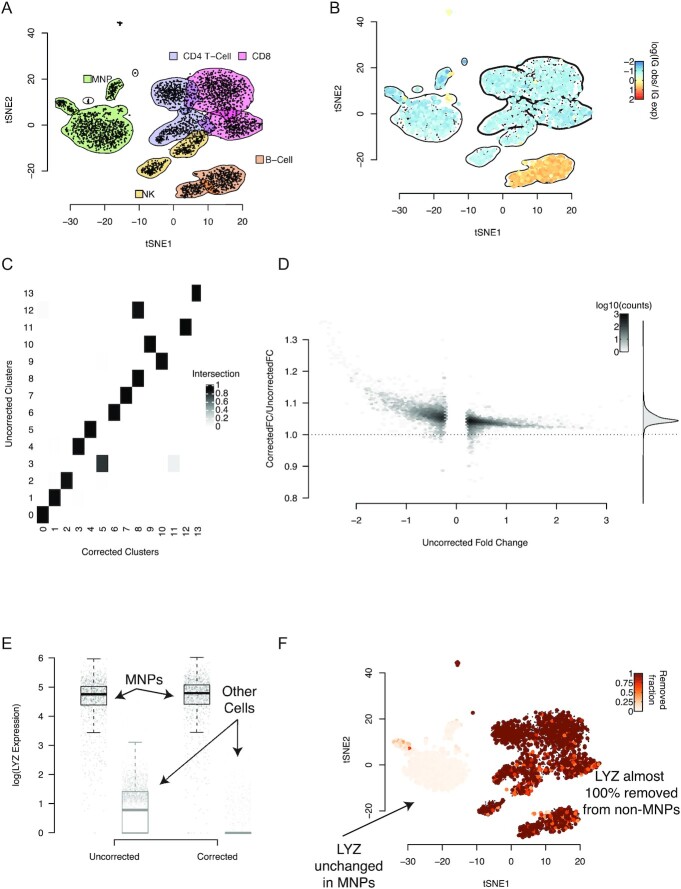
The PBMC dataset and how it changes when background correction is applied. **A**, A tSNE representation of the data, with cluster boundaries shown by density contours and shaded according to the cell type they represent. MNP: mononuclear phagocytes; NK: natural killer cells. **B**, The same tSNE representation, but cells are now coloured by their rate of expression of immunoglobulin (IG) genes compared to the rate at which IG is expressed in the background on a log_10_ scale. Positive values correspond to higher IG expression in a cell than in the background, with values significantly >0 only possible if the cell endogenously expresses IG. The density contours of the clusters with no cell that endogenously expresses IG (as determined by a Poisson test) are marked in boldface and used to estimate the global contamination ratio. **C**, The fraction of cells shared between clusters determined with the same parameters before and after application of SoupX. **D**, The improvement in marker specificity following application of SoupX. All genes that are markers of a cluster either before or after correction are identified and their expression log fold change (FC) relative to the clusters they do not mark is calculated before and after correction. The y-axis of this plot shows the fractional change in log FC after applying SoupX for all genes. Genes are grouped into bins for ease of representation, with the number of genes in each bin given by the colour scale. The marginal distribution across all genes is shown on the right and the dotted line corresponds to no change in marker specificity after correction. **E**, The improvement in marker sensitivity for the gene *LYZ*, which is a marker for mononuclear phagocytes (MNPs). The corrected and uncorrected expression levels are shown split by cells labelled as MNPs and all others. **F**, This same change in expression shown on the tSNE map, where the colour scale represents the fraction of *LYZ* expression that has been removed by SoupX.

Next we tested our method on a dataset consisting of PBMCs, measured in a single channel [2]. We used the Seurat package [18,19] to produce a *t*-distributed stochastic neighbour embedding (tSNE) representation of the data and annotated clusters of cells based on the expression of canonical marker genes (Fig. 3A).

Applying the automated procedure (see Supplementary Methods) to estimate the contamination fraction produced a background contamination rate of $6\%$. To confirm the accuracy of this estimate, we also calculated the background contamination rate using a set of genes that could be assumed to be unexpressed in some cells (i.e., where *m_g, c_* = 0).

To aid appropriate selection of such a gene set, we reasoned that the ideal genes for estimating the contamination rate would be ubiquitously present at a low level in all droplets due to high expression in the ambient RNA. They would also be present at a high level when a cell endogenously expresses the gene, allowing us to unambiguously separate droplets with endogenously expressing cells (i.e., where *m_g, c_* > 0) from those where the expression is solely due to contamination (*m_g, c_* = 0).

Based on this reasoning, we developed a heuristic that ranks the 500 genes with the highest expression in the background by their bimodality of expression across all droplets in a channel. A plot based on applying this heuristic to the PBMC data shows the expression distribution across all cell-containing droplets in the dataset (Supplementary Fig. S5). This heuristic suggests that immunoglobulin (IG) genes, such as *IGKC* and *IGLC2*, are both highly expressed in the soup and highly specific in their expression, making them good candidates for estimating the contamination fraction in this dataset.

To select a precise set of cells for which we could use IG genes to estimate the contamination, we identified all cells whose IG expression was significantly greater than in the background contamination (Poisson test, false discovery rate 0.05; Supplementary Materials). These represent cells endogenously expressing IG. We only used cells from clusters with no cells identified as endogenously expressing IG to estimate the contamination rate (Fig. 3B). For the PBMC data, this identified IG expression in T cells as purely due to contamination and calculated a background contamination rate of $\sim 5\%$.

Having calculated the global contamination rate for the PBMC data, we then corrected the PBMC data for background mRNA contamination and re-analysed the data with Seurat using the same settings. Comparing cluster membership before and after correction revealed that the same number of clusters was identified, but some cells changed which cluster they belonged to (Fig. 3C).

Next we identified marker genes for each cluster in both the corrected and uncorrected PBMC data using a Wilcoxon rank sum test and calculated the expression fold change between the cluster and all other cells. We compared the fold changes for the same genes in the same clusters before and after correction and found that correction for background contamination systematically increased the fold change contrast for marker genes (Fig. 3D). That is, correction for background contamination made marker genes more specific to the cluster they were markers of. Furthermore, additional genes were found as markers in the corrected data that were not identified in the uncorrected data.

As a specific example, we found that correction of ambient RNA contamination changes the pattern of expression of *LYZ* in the PBMC data (Fig. 3E and F). This improved the specificity of *LYZ* as a marker gene for mononuclear phagocytes (MNPs) (Fig. 3E) by removing its expression from all other cell types, while leaving its expression in MNPs unchanged (Fig. 3F).

## Ambient RNA Confounds Interpretation in Complex Experiments

**Figure 4:**
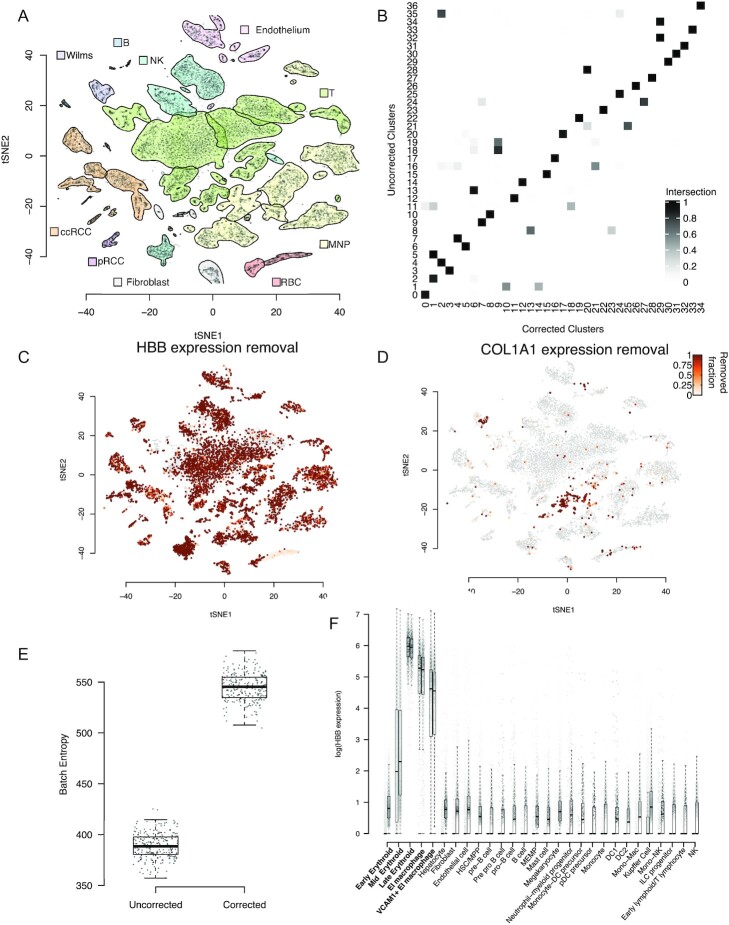
The application of SoupX to complex, multi-channel data. **A**, A tSNE representation of the data, with cluster boundaries shown by density contours and shaded according to the cell type they represent. ccRCC: clear-cell renal cell carcinoma cells; pRCC: papillary cell renal cell carcinoma cells; RBC: red blood cells; MNP: mononuclear phagocytes. **B**, The fraction of cells shared between clusters determined with the same parameters before and after application of SoupX. **C**, The improvement in marker sensitivity for the gene *HBB*, which is a marker for red blood cells. The colour scale represents the fraction of *HBB* expression that has been removed by SoupX. **D**, Same as **C** but for *COL1A1*. **E**, The cross-batch entropy before and after SoupX has been applied. The entropy measures the level of local mixing (100 nearest neighbours) for 100 cells selected from each cluster [20]. **F**, The distribution of *HBB* expression (y-axis, log scale) in the fetal liver data by cell type (x-axis), with the erythroid lineage marked in boldface. For each cell type, the expression distribution is shown before (right) and after (left) application of SoupX. Dots represent individual cells and box plots show the distribution of expression values where the central line is the median, box boundaries are the first and third quartiles, and the whiskers extend to 1.5 times the interquartile range.

As a further test of the biological utility of our method we considered an experiment combining 7 kidney tumours processed across 10 channels (Supplementary Table S1). As with the PBMCs, we analysed corrected and uncorrected data using the Seurat package; Fig. 4A shows a tSNE plot of the uncorrected data. Haemoglobin genes were used to estimate the contamination fraction in most channels (Supplementary Fig. S10). This choice of gene set for estimating contamination was motivated by the ubiquitous presence of red blood cells (with red cell lysis forming part of the tissue treatment protocol) in these samples, together with the knowledge that haemoglobin genes are highly specific to red blood cells. We compared the resulting estimates of the contamination fraction with those obtained by applying the automated method and found good agreement (Supplementary Fig. S6).

Applying SoupX and re-analysing the kidney tumour data revealed that, in contrast to the PBMC data, many cells changed cluster and with the same clustering parameters 2 fewer clusters were identified in the corrected data (Fig. 4B). Furthermore, we found that the expression ratio of marker genes between the cluster they mark and all other cells increased systematically after correction for background contamination (Supplementary Fig. S7).

We found that the correction of background contamination changed the distribution of expression of many genes across cells in a way that would alter the biological interpretation. For example, while it is unlikely to be biologically misinterpreted, SoupX completely removes the expression of haemoglobin genes from all cells except red blood cells (Fig. 4C).

In other cases, the misattribution of gene expression to cell types that do not truly express them could lead to false conclusions. An example of this is the cluster of T and MNPs in Fig. 4A and D, which express the collagen genes *COL1A1*, *COL1A2*, and *COL3A1* before background correction. The expression of collagen genes might be interpreted as evidence that the leukocytes are resident in the tissue. However, our method identifies that a high fraction of this expression is due to contamination (Fig. 4D).

Because the ambient mRNA expression profile is experiment specific, we reasoned that background contamination likely creates batch effects. That is, 2 identical cells captured in different experiments will appear different owing to differences in their cell-free RNA composition. We therefore calculated the cross-batch entropy of the kidney tumour data before and after background correction [20]. This analysis shows that the batch-mixing entropy is increased after background correction, indicating better mixing between samples (Fig. 4E).

As a further example of the biological utility of SoupX, we applied SoupX to 40 channels of human fetal liver data (Supplementary Fig. S8). Before correction for background contamination, a large number of cells outside the erythroid (red blood cell) lineage express erythroid markers such as *HBB* in combination with other cell type markers. This widespread expression of multiple distinct markers could potentially indicate the presence of doublets. Application of SoupX allows this explanation to be ruled out, showing that *HBB* is only truly expressed in erythroid cell types (Fig. 4F).

Application of SoupX is also able to identify those cell types where biologically unexpected combinations of genes represent genuine biological phenomena. One example of this is the expression of the erythroid gene *GYPA* in the EI macrophage populations, which could be the consequence of either contamination or a biological phenomenon. Application of SoupX confirms that this expression represents genuine gene expression and not ambient RNA contamination (Supplementary Fig. S9).

## Discussion

We have shown that cell-free RNA is omnipresent in droplet-based scRNA-seq data and have proposed a method to identify, quantify, and remove its contaminating effect. We find that accounting for contamination improves the specificity of marker genes, identifies new markers, and is essential for the correct biological interpretation of complex experiments.

We have shown some potential misinterpretations of kidney tumour and fetal liver data driven by ambient mRNA contamination, but examples are sure to abound in other tissues. For instance, in endocrine tissues, it is crucial to understand which cell types secrete a particular hormone. The misassigned expression of even a single hormone gene can fundamentally change how investigators think about a cell type. Such problems will become increasingly common as efforts to compare similar cell types across tissues progress.

The best case for applying SoupX occurs when the user can specify a set of genes and cells where there is no cell endogenous expression, i.e., a set of genes and cells where it is safe to assume that the only source of expression for these genes is from background contamination. The expectation is that biological knowledge of the experiment being performed will guide this choice. Where such a set of genes and cells can be provided, this will yield the best results.

For example, solid-tissue experiments are frequently highly contaminated with red blood cells and red cell lysis is used to prepare the samples [15]. As such, haemoglobin genes are often ubiquitously present in the background. Furthermore, red blood cells are the only cells that produce haemoglobin under normal physiological conditions, so for the set of haemoglobin genes, it is safe to assume that there is no cell endogenous expression for cells that are not red blood cells. Finally, red blood cells express haemoglobin genes in such extreme abundance that they can be trivially identified by comparing the ratio of observed haemoglobin genes to that present in the background contamination (Supplementary Fig. S10). These properties make haemoglobin genes a sensible choice for most solid-tissue experiments.

Heuristics, such as the bimodal expression ranking in Supplementary Fig. S5, can help aid biologically motivated gene selection. However, we recognize that selecting an appropriate set of genes to estimate contamination will not always be possible. To address this issue, we include an automated contamination estimation procedure. By using all high-quality marker genes identified in the data to independently estimate the contamination fraction, this method estimates the true contamination fraction by assuming that inaccurate estimates of the contamination fraction are not strongly correlated (i.e., there is no preferred, incorrect estimate). We show that this automation gives comparable results to the manual method. Although this procedure requires cells to be clustered, clustering information is used primarily to identify marker genes. As such, consistent estimates of the contamination fraction will be obtained for any sensible clustering of the data.

It is also possible to manually specify the contamination fraction, which can be useful when the aforementioned estimation procedures are deemed inaccurate or it is desirable to overcorrect the data. For most applications, the consequences of manually setting an unrealistically high contamination rate are likely to be minimal. Contamination is preferentially removed from genes closest to the background expression (i.e., genes with low levels of expression), meaning that setting a higher global contamination rate is unlikely to completely remove the expression of genes that are truly markers of a cell. Thus in some applications it may be preferable to overcorrect for background contamination and remove a small amount of genuine signal to ensure that all the background contamination has been removed. We also find that our method is robust to small inaccuracies in the estimation of the global contamination rate (Supplementary Fig. S4).

Since SoupX was first released, several other tools have been developed that aim to remove background contamination. SoupOrCell [21] uses the identification of conflicting genotypes to identify ambient RNA contamination, limiting its application to mixed-genotype experiments. Cell Bender [22] uses a deep generative model to estimate shared expression patterns likely to represent distinct cell types while simultaneously removing contamination. This deep generative model comes with a heavy computational cost compared to other tools, and the output of the model (which in effect estimates *m_g, c_* for each cell type) provides an imputed cell profile rather than raw counts with the background “subtracted off,” which SoupX provides. Finally, DecontX [23] relies on accurate clustering of the data to estimate and remove the background without the need for gene counts from empty droplets. This allows DecontX to be applied when empty droplet counts are not available but also means that the results are potentially heavily dependent on the accuracy of the clustering provided. By contrast, SoupX can be applied generally, is computationally inexpensive, and does not depend heavily on accurate pre-annotation of input data.

To make our method easily applicable, we provide an R package, SoupX, which can be used to estimate and remove ambient mRNA contamination. This package is available on the Comprehensive R Archive Network (CRAN) and is provided with a vignette to assist the user in understanding how best to apply the method. The output of the SoupX package is a corrected table of counts, which can be used as input for standard workflows, and running SoupX does not add appreciably to the computational cost of standard single-cell analyses. We envision background correction forming a standard part of droplet-based scRNA-seq analysis pipelines.

## Data Availability

The 10X species-mixing dataset was the mixture of the human cell line 293T and the mouse cell line 3T3 described in [2]. We used the data mapped and quantified using Cell Ranger 1.1.0 from https://support.10xgenomics.com/single-cell-gene-expression/datasets/1.1.0/293t_3t3. The DropSeq species-mixing data were obtained from [14], specifically SRR1748411. The PBMC data were taken from [2]. The kidney tumour dataset was taken from [15]. The fetal liver data [16] are available from ArrayExpress with accession code E-MTAB-7407. The mapped datasets supporting the results of this article are available in the GigaDB repository [24].

## Availability of Supporting Code and Requirements

Project name: SoupX

Project home page: https://github.com/constantAmateur/SoupX

Operating systems: Platform indepdent

Programming language: R

Other requirements: R 3.5.0 or higher

License: GNU GPL

RRID: https://scicrunch.org/browse/resources/SCR_019193

biotools ID: soupx

The SoupX R package is also available from CRAN at https://github.com/constantAmateur/SoupX, the scripts to reproduce this analysis are at https://github.com/constantAmateur/ambientRNA_paper, and a Docker image containing all code and data needed to generate the results in this article can be obtained from https://hub.docker.com/r/constantamateur/soupxpaper.

## Additional Files

**Supplementary Table S1**. Sample information for the different datasets used in this article.

**Supplementary Table S2**. Pearson correlation coefficient between the background contamination profile and all cells in a channel averaged, after removing the genes above the 99th expression quantile.

**Supplementary Figure S1**. Schematic illustrating the procedure used to estimate the global contamination rate using the gene *IGHD* on the PBMC data. On the left, individual cells are marked red when their expression of *IGHD* is higher than would be possible even if the cell were nothing but contamination. That is, cells where *IGHD* must be endogenously expressed are marked red. Any cluster containing such a cell is excluded, and the global contamination fraction is estimated using cells in the remaining clusters (right of plot).

**Supplementary Figure S2**. The correlation between "true background," which is defined by aggregating across mouse transcripts in human cells and vice versa, with the background expression profile derived using only droplets. Total number of UMIs is given on the x-axis.

**Supplementary Figure S3**. The ratio of human to mouse transcripts on a log_10_ scale (y-axis) for all droplets in the DropSeq species-mixing experiment. Droplets containing cells are marked in black. The x-axis gives the average number of UMIs between human and mouse for each cell.

**Supplementary Figure S4**. The x-axis gives the true contamination rate measured using the cross-species transcripts in each cell. The y-axis gives the effective contamination rate obtained by applying SoupX at the cluster level using a constant global contamination rate, calculated as the fraction of removed counts by the application of SoupX. The line shows perfect correlation, and red and blue dots represent the 10X and DropSeq species-mixing experiments, respectively.

**Supplementary Figure S5**. Distribution of expression relative to background for genes in the PBMC data. The red line indicates the global estimate of the contamination fraction that would be obtained if just that gene were used to estimate contamination. Genes that are most useful for contamination estimation have a bimodal distribution, with cells genuinely expressing the gene yielding a value on the y-axis >0 and cells that do not express the gene having a value clustered around the true contamination rate.

**Supplementary Figure S6**. Comparison of the contamination fraction estimated by the automated method (x-axis) and by manually supplying a gene set (y-axis), for each channel in the kidney tumour data. The dashed line indicates perfect correlation, and the Pearson correlation is shown in the upper left.

**Supplementary Figure S7**. The improvement in marker specificity following application of SoupX to the kidney tumour data. Note the different scale of the y-axis compared to Fig. 3. All genes that are markers of a cluster either before or after correction are identified, and their expression log fold change (FC) relative to the clusters that they do not mark is calculated before and after correction. The y-axis of this plot shows the fractional change in log FC after applying SoupX for all genes. Genes are grouped into bins for ease of representation, with the number of genes in each bin given by the colour scale. The marginal distribution across all genes is shown on the right, and the dotted line corresponds to no change in marker specificity after correction.

**Supplementary Figure S8**. Uniform manifold approximation and projection (UMAP) representation of the single-cell fetal data. Each point is coloured by its cell type and a cell type label is placed at the position of the average cell.

**Supplementary Figure S9**. Normalized gene expression of *GYPA* (y-axis) in fetal liver data by cell type before and after ambient RNA removal by SoupX (x-axis). The cell types on the x-axis represent the different cell types as annotation in Supplementary Fig. S8. For each cell type, box plots indicate the median, quartiles, and 1.5 times the interquartile range for cells after SoupX correction (left) and before (right). For each distribution, each cell's expression is also shown with horizontal jitter and transparency inversely proportional to the number of cells of that type. The 2 EI Macrophage populations are emphasized in boldface.

**Supplementary Figure S10**. The fractional expression of haemoglobin genes in each cell, relative to the rate of expression in the background in 1 of the kidney tumour channels. This fraction is given by the colour of each point on a log scale. Points that have been determined to not endogenously express haemoglobin genes are marked with a green outline. The x- and y-axis are the tSNE coordinates supplied by cellranger for this channel.

**Supplementary Methods**.A more verbose description of the SoupX method and details of data processing of the different datasets used in this article.

## Abbreviations

IG: immunoglobulin; MNP: mononuclear phagocyte; mRNA: messenger RNA; PBMC: peripheral blood mononuclear cell; scRNA-seq: single-cell RNA sequencing; SRA: Sequence Read Archive; tSNE: t-distributed stochastic neighbour embedding; UMI: unique molecular identifier.

## Competing Interests

The authors declare that they have no competing interests.

## Funding

We acknowledge funding from Wellcome, Sam Behjati fellowship, and core funding to the Sanger Institute.

## Authors' Contributions

M.D.Y. conceived the project, developed the method, and wrote the manuscript. S.B. contributed to the method development.

## Supplementary Material

giaa151_GIGA-D-20-00034_Original_Submission

giaa151_GIGA-D-20-00034_Revision_1

giaa151_GIGA-D-20-00034_Revision_2

giaa151_Response_to_Reviewer_Comments_Original_Submission

giaa151_Response_to_Reviewer_Comments_Revision_1

giaa151_Reviewer_1_Report_Original_Submission Shreejoy Tripathy -- 2/18/2020 Reviewed

giaa151_Reviewer_2_Report_Original_Submission Chris L Plaisier, Ph.D -- 2/21/2020 Reviewed

giaa151_Supplemental_Figures_and_Tables
