# Supplementary material for: SoupX removes ambient RNA contamination from droplet-based single-cell RNA sequencing data
Source: Gigascience. 2020 Dec 26;9(12):giaa151. doi: 10.1093/gigascience/giaa151 (PMC7763177; doi:10.1093/gigascience/giaa151)
Supplement: giaa151_GIGA-D-20-00034_Revision_1 [file giaa151_giga-d-20-00034_revision_1.pdf]

GigaScience

SoupX removes ambient RNA contamination from droplet-based single-cell RNA sequencing data

--Manuscript Draft--

**Manuscript Number:**	GIGA-D-20-00034R1
**Full Title:**	SoupX removes ambient RNA contamination from droplet-based single-cell RNA sequencing data
**Article Type:**	Research
**Funding Information:**	
**Abstract:**	Background Droplet based single-cell RNA sequence analyses assume all acquired RNAs are endogenous to cells. However, any cell free RNAs contained within the input solution are also captured by these assays. This sequencing of cell free RNA constitutes a background contamination that confounds the biological interpretation of single-cell transcriptomic data. Results We demonstrate that contamination from this ‘soup’ of cell free RNAs is ubiquitous, with experiment-specific variations in composition and magnitude. We present a method, SoupX, for quantifying the extent of the contamination and estimating ‘background corrected’ cell expression profiles that seamlessly integrate with existing downstream analysis tools. Applying this method to several datasets using multiple droplet sequencing technologies, we demonstrate that its application improves biological interpretation of otherwise misleading data, as well as improving quality control metrics. Conclusions We present ‘SoupX’, a tool for removing ambient RNA contamination from droplet based single cell RNA sequencing experiments. This tool has broad applicability and its application can improve the biological utility of existing and future data sets.
**Corresponding Author:**	Matthew Daniel Young<br>Wellcome Trust Sanger Institute<br>Cambridge, UNITED KINGDOM
**Corresponding Author Secondary Information:**	
**Corresponding Author's Institution:**	Wellcome Trust Sanger Institute
**Corresponding Author's Secondary Institution:**	
**First Author:**	Matthew Daniel Young
**First Author Secondary Information:**	
**Order of Authors:**	Matthew Daniel Young
	Sam Behjati
**Order of Authors Secondary Information:**	
**Response to Reviewers:**	Dear Dr. Hans Zauner,<br><br>Please find attached our revised manuscript. As previously discussed, in this revision we have included additional data derived from a different biological context (human fetal liver) to further demonstrate the biological utility of SoupX. To ensure that this demonstration of utility is well motivated, we focus on issues raised during peer review of the fetal liver paper, which SoupX is able to definitively resolve. Also as requested, we have included a discussion of how SoupX relates to other ambient mRNA correction tools that have been published since SoupX was first written.<br><br>We provide a point by point response to the points raised by the original reviewers and Dr. Varodom Charoensawan in the attached supplementary document. Although we do not agree with all the points raised by reviewers, the process has undoubtedly improved the manuscript. Thank you for your patience with the further COVID associated delay and we hope that you agree that our revised manuscript adequately addresses the remaining concerns with our manuscript.

Powered by Editorial Manager® and ProduXion Manager® from Aries Systems Corporation

	With best wishes,<br><br>Matthew Young and Sam Behjati
**Additional Information:**	
**Question**	**Response**
Are you submitting this manuscript to a special series or article collection?	No
**Experimental design and statistics**<br><br>Full details of the experimental design and statistical methods used should be given in the Methods section, as detailed in our Minimum Standards Reporting Checklist. Information essential to interpreting the data presented should be made available in the figure legends.<br><br>Have you included all the information requested in your manuscript?	Yes
**Resources**<br><br>A description of all resources used, including antibodies, cell lines, animals and software tools, with enough information to allow them to be uniquely identified, should be included in the Methods section. Authors are strongly encouraged to cite Research Resource Identifiers (RRIDs) for antibodies, model organisms and tools, where possible.<br><br>Have you included the information requested as detailed in our Minimum Standards Reporting Checklist?	Yes
**Availability of data and materials**<br><br>All datasets and code on which the conclusions of the paper rely must be either included in your submission or deposited in publicly available repositories (where available and ethically appropriate), referencing such data using	Yes

a unique identifier in the references and in the “Availability of Data and Materials” section of your manuscript.<br><br>Have you have met the above requirement as detailed in our Minimum Standards Reporting Checklist?	

Powered by Editorial Manager® and ProduXion Manager® from Aries Systems Corporation

Revised manuscript

Click here to access/download;Manuscript;manuscriptRevision2.pdf

OXFORD (GIGA)n SCIENCE

GigaScience, 2020, 1–21

doi: xx.xxxx/xxxx
Manuscript in Preparation
Paper

PAPER

SoupX removes ambient RNA contamination from droplet based single-cell RNA sequencing data

Matthew D Young[1,*] and Sam Behjati[1,2,3]

[1]Wellcome Trust Sanger Institute and [2]Cambridge University Hospitals NHS Foundation Trust and [3]Department of Paediatrics, University of Cambridge

*my4@sanger.ac.uk

Abstract

Background Droplet based single-cell RNA sequence analyses assume all acquired RNAs are endogenous to cells. However, any cell free RNAs contained within the input solution are also captured by these assays. This sequencing of cell free RNA constitutes a background contamination that confounds the biological interpretation of single-cell transcriptomic data. **Results** We demonstrate that contamination from this 'soup' of cell free RNAs is ubiquitous, with experiment-specific variations in composition and magnitude. We present a method, SoupX, for quantifying the extent of the contamination and estimating 'background corrected' cell expression profiles that seamlessly integrate with existing downstream analysis tools. Applying this method to several datasets using multiple droplet sequencing technologies, we demonstrate that its application improves biological interpretation of otherwise misleading data, as well as improving quality control metrics. **Conclusions** We present 'SoupX', a tool for removing ambient RNA contamination from droplet based single cell RNA sequencing experiments. This tool has broad applicability and its application can improve the biological utility of existing and future data sets.

Key words: scRNA-seq; Decontamination; Pre-processing

Introduction

Droplet based single-cell RNA sequencing (scRNA-seq) has enabled quantification of the transcriptomes of hundreds of thousands of cells in single experiments [1, 2]. This technology underpins recent advances in understanding normal and pathological cell behaviour [3, 4, 5, 6, 7, 8]. Moreover, large scale efforts to create a 'Human Cell Atlas' critically depend on the accuracy and cellular specificity of the transcriptional readout produced by droplet based scRNA-seq [9, 10].

A core assumption of droplet based scRNA-seq is that each droplet, within which molecular tagging and reverse transcription take place, contains mRNA from a single-cell. Violations of this assumption, which may distort the interpretation of scRNA-seq data, are common in practice. Clear examples include droplets that contain multiple cells (doublets), and empty droplets. Attempts to detect and remove doublets are an active area of research [11, 12, 13].

Another phenomenon that violates this assumption is the sequencing of cell free RNA from the input solution, admixed with a cell in its enclosing droplet. It is recognised that these contaminating non-endogenous RNAs are present even within data sets of the highest quality [2]. Here, we show that this 'soup' of cell free mRNAs is ubiquitous and non-negligible in magnitude. Since the character and extent of ambient mRNA contamination varies by experiment, with increased contamination in necrotic or complex samples, ambient mRNAs may significantly confound the biological interpretation of scRNA-seq data. We present SoupX, a method for quantifying the extent of ambient mRNA contamination whilst purifying the true, cell specific signal from the observed mixture of cellular and exogenous mRNAs.

In this paper we begin by briefly describing the SoupX method. Following this we consider a range of datasets, summarised in Table S1. We first investigate two "species mixing" datasets run on the Chromium 10X [2] and DropSeq [14] platforms, which allow us to directly identify contaminating mRNAs and test our method's accuracy. We then demonstrate how SoupX can be applied in practice using a dataset of peripheral blood mononuclear cells (PBMCs) [2].

Compiled on: October 13, 2020.
Draft manuscript prepared by the author.

Key Points

- The signal from droplet based single cell RNA sequencing is ubiquitously contaminated by capture of ambient mRNA.
- SoupX is a method to quantify the abundance of these ambient mRNAs and remove them.
- Correcting for ambient mRNA contamination improves biological interpretation.

We further explore the biological benefits of SoupX using a complex 'kidney tumour' dataset, which consists of 12 kidney tumour biopsies [15]. As a final test, we apply our method to human fetal liver data [16]. We conclude with some general remarks about ambient RNA contamination, others tools to correct for its effect, and the consequences of failing to account for ambient RNAs presence.

The SoupX method

Droplet based scRNA-seq methods produce counts of unique molecular identifiers (UMIs) for genes in thousands of cells. The aim of a scRNA-seq experiment is to infer the number of molecules present for each type of gene within each cell from this data. However, the observed counts arise from a mixture of mRNAs produced by the captured cell and those present due to background contamination. SoupX aims to remove the contribution of the cell free mRNA molecules from each cell and recover the true molecular abundance of each gene in each cell.

The algorithm consists of the following three steps (summarised in Figure 1):

i. Estimate the ambient mRNA expression profile from empty droplets.
ii. Estimate (or manually set) the contamination fraction, the fraction of UMIs originating from the background, in each cell.
iii. Correct the expression of each cell using the ambient mRNA expression profile and estimated contamination.

SoupX produces a modified table of counts, which can be used in place of the original count matrix in any downstream analysis tool.

To estimate the background expression profile we consider all droplets with fewer than N_{emp} UMIs, which we assume unambiguously do not contain cells. The fraction of background expression from gene g, b_g is then given by,

$$b_g = \frac{\sum_d n_{g,d}}{\sum_d \sum_g n_{g,d}} \tag{1}$$

where $n_{g,d}$ is the number of counts for gene g in droplet d and the sum over d is taken over all droplets with fewer than N_{emp} UMIs (Figure 1). The species mixing experiment allows us to compare how accurately b_g recapitulates the true background expression found within each cell, revealing that any value of $N_{emp} < 100$ produces a good correlation, with the best correlation given when $N_{emp} < 10$ (Figure S2).

The most challenging part of using SoupX is estimating or specifying the number of UMIs in each cell that are contributed by background contamination. In general, the observed number of UMIs for gene g in cell c is given by,

$$n_{g,c} = m_{g,c} + o_{g,c} \tag{2}$$

where $m_{g,c}$ are the cell endogenous counts and $o_{g,c}$ are the counts from the background. We assume the relative abundance of genes that make up the background does not differ between cells, which allows us to write,

$$o_{g,c} = N_c \rho_c b_g \tag{3}$$

where $N_c = \sum_g n_{g,c}$, and ρ_c is the background contamination fraction. In general $m_{g,c}$ is unknown and what we are aiming to measure. To proceed, we assume that there a combination of genes and cells for which $m_{g,c} = 0$ exists. The genes for which $m_{g,c} = 0$ for a given cell are those genes that are strong negative markers of the cell type c. For example, the gene *HBB* is a strong positive marker for erythroid cells (red blood cells), but should not be expressed in any other cell type. So for any cell c which is not an erythroid cell, *HBB* will not be expressed (i.e., $m_{HBB,notErythroid} = 0$).

Given a set of genes/cells for which we can assume there is no cell endogenous expression (i.e., $m_{g,c} = 0$) we calculate the cell specific contamination fraction,

$$\rho_c = \frac{\sum_g n_{g,c}}{N_c \sum_g b_g} \tag{4}$$

where the sum is taken across all genes in cell c for which it is assumed $m_{g,c} = 0$. SoupX optionally uses clustering information to refine the set of cells for which it can be assumed that $m_{g,c} = 0$. If it can be shown for any cell c in cluster P that $m_{g,c} > 0$, then it is assumed that $m_{g,c} > 0$ for all $c \in P$ (see Figure S1 and Section).

If known from prior biological knowledge, the set of genes/cells for which it can be assumed that $m_{g,c}$ can be provided as input to SoupX. Where this is not known in advance, we provide an automated alternative to estimate the contamination fraction (see Figure 1 and Section). The automated approach first identifies markers of each cluster of cells in the data. For each strong marker, it is assumed that $m_{g,c} = 0$ for all cells in clusters where the gene is not a marker and the contamination fraction is estimated (Figure S1). Performing this estimation across all strong marker genes provides a set of estimates of the contamination fraction. To obtain a final value, it is assumed that inaccurate estimates will have no preferred value while true estimates will cluster around the true value. The most common values is taken as the final estimate of the contamination fraction (see Figure 1, step 2.2).

Having determined the contamination fraction ρ_c and the background expression profile b_g, the cell endogenous counts are intuitively given by,

$$m_{g,c} = n_{g,c} - N_c \rho_c b_g \tag{5}$$

where $n_{g,c}$ are the observed counts, $N_c = \sum_g n_{g,c}$, and b_g and ρ_c are calculated as described above.

Although the intuition of Equation 5 is correct, in practice $m_{g,c}$ is estimated by maximising a multinomial likelihood as described in Section . This procedure is further enhanced when cluster assignments are given, by performing the correction on counts aggregated at the cluster level, then distributing the corrected counts between cells in the cluster in proportion to their size (see Figure 1). This additional step helps overcome the sparsity of scRNA-seq data, which would otherwise make it impossible to distinguish a single count due to contamination from a single count due to endogenous expression in many circumstances.

The estimated value of $m_{g,c}$ can then be used in place of $n_{g,c}$ in any downstream analysis.

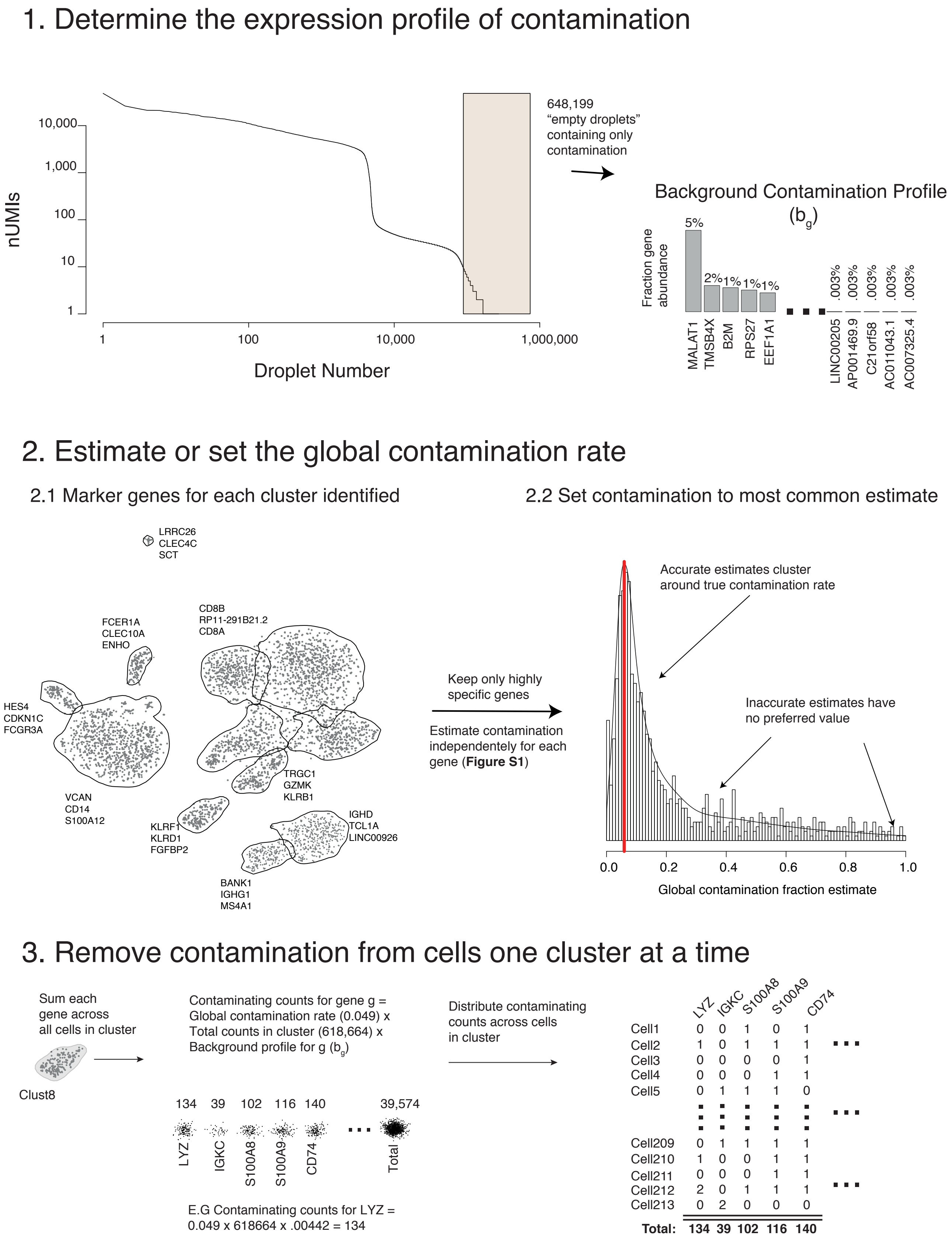

Figure 1. A visual summary of the SoupX method, using data from the PBMC dataset.

Properties of ambient RNA

We next investigate the properties of ambient RNA contamination in data where ground truth is available, the "species mixing" experiments combining mouse and human cell lines using 10X [2] and Drop-Seq technologies [14]. Figure 2A shows the relative abundance of human and mouse mRNAs in each droplet in the 10X data. Droplets containing human (top-right) and mouse (bottom-right) cells show that ∼ 1% of observed transcripts are cross species contamination. This rate of cross species contamination provides a lower bound on the total rate of ambient mRNA contamination as there will also be an additional contribution due to contaminating mRNAs from the same species (we later show the true contamination rate is $\sim 2\%$). A similar effect is seen in the Drop-Seq based species mixing data (Supplementary Figure S3). These observations demonstrate that cell free mRNA contamination is present even in highly controlled experiments.

To investigate the composition of cell free mRNAs, we compared the aggregate expression profile of all droplets containing cells to all droplets with ≤ 10 UMIs, which we assumed to contain only ambient mRNAs. These two profiles were highly correlated in the 10X species experiment (Figure 2B) with a high correlation found in all other datasets considered (Pearson correlation 0.71 to 0.96, median 0.86; Table S2). The strength of the correlation implies that cell free contamination represents an approximately uniform sampling of the cells in the sequencing batch (i.e., channel).

Next we estimated the contamination fraction, the fraction of expression derived from the cell free mRNA background in each cell. In each cell we identify a set of genes that must have originated from the ambient mRNA; human transcripts in mouse cells and visa-versa. For these genes/cells it is assumed that $m_{g,c} = 0$ and the contamination fraction is calculated using Equation 4. Figure 2C reveals that there is little variation in the contamination fraction within a channel, in both the 10X and DropSeq data.

In most experiments there is less power to determine cell-specific contamination fractions and so SoupX assumes a constant contamination fraction within a channel. When clustering information is provided, the redistribution of counts from cluster level to individual cells, automatically removes more counts from contaminated cells, even when only a global estimate of the contamination is given (Figure S4; Section). Where a cell specific expression estimate is needed, SoupX employs a hierarchical bayes method to share information between cells (Section).

It may be hypothesised that the absolute number of contaminating mRNA molecules is the quantity that is approximately constant and that the contamination should vary with the number of mRNA molecules contributed by the captured cell. That is, that contamination fraction should vary as a function of cellular mRNA contribution, with the number of detected UMIs being a proxy for this. Consistent with this, Figure 2C shows that the greatest contamination occurs in droplets with the fewest UMIs. However, the contamination fraction is still approximately constant across most of the UMI range. This is likely a consequence of the fact that the capture efficiency of molecules in droplet based experiments varies by as much as an order of magnitude [17]. Thus variation due capture efficiency is likely to swamp variation due to "cell size" in most experiments, making constant contamination fraction a reasonable approximation.

To test the accuracy of SoupX in removing contaminating counts while retaining those due to endogenous expression we compared the fraction of expression from cross-species and within-species genes before and after SoupX contamination correction. This analysis revealed (Figure 2D) that mouse expression in human cells (and visa-versa) was decreased by at least a factor of two and usually an order of magnitude by the SoupX contamination removal in both 10X and DropSeq experiments. By contrast the fraction of expression derived from genes corresponding to the correct species was effectively unchanged for all cells.

Application of SoupX to PBMC data

Next we tested our method on a data set consisting of peripheral blood mononuclear cells (PBMCs), measured in a single channel [2]. We used the Seurat package [18, 19] to produce a tSNE representation of the data and annotated clusters of cells based on the expression of canonical marker genes (Figure 3A).

Applying the automated procedure (see Section) to estimate the contamination fraction produced a background contamination rate of 6%. To confirm the accuracy of this estimate, we also calculated the background contamination rate using a set of genes that could be assumed to be unexpressed in some cells (i.e., where $m_{g,c} = 0$).

To aid appropriate selection of such a gene set, we reasoned that the ideal genes for estimating the contamination rate would be ubiquitously present at a low level in all droplets due to high expression in the ambient RNA. They would also be present at a high level when a cell endogenously expresses the gene, allowing us to unambiguously separate droplets with endogenously expressing cells (i.e., where $m_{g,c} > 0$) from those where the expression is solely due to contamination ($m_{g,c} = 0$).

Based on this reasoning, we developed a heuristic that ranks the 500 genes with the highest expression in the background by their bimodality of expression across all droplets in a channel. A plot based on applying this heuristic to the PBMC data shows the expression distribution across all cell containing droplets in the dataset (Figure S5). This heuristic suggests that immunoglobulin genes, such as *IGKC* and *IGLC2*, are both highly expressed in the soup and highly specific in their expression, making them good candidates for estimating the contamination fraction in this dataset.

To select a precise set of cells for which we could use immunoglobulin genes (IG genes) to estimate the contamination, we identified all cells whose IG expression was significantly greater than in the background contamination (Poisson test, FDR 0.05; Section). These represent cells endogenous expressing IG. We only used cells from clusters with no cells identified as endogenously expressing IG to estimate the contamination rate (Figure 3B). For the PBMC data, this identified IG expression in T cells as purely due to contamination and calculated a background contamination rate of $\sim 5\%$.

Having calculated the global contamination rate for the PBMC data, we then corrected the PBMC data for background mRNA contamination and re-analysed the data with Seurat using the same settings. Comparing cluster membership before and after correction revealed that the same number of clusters was identified, but some cells changed which cluster they belonged to (Figure 3C).

Next we identified marker genes for each cluster in both the corrected and uncorrected PBMC data using a Wilcoxon Rank Sum test and calculated the expression fold change between the cluster and all other cells. We compared the fold changes for the same genes in the same clusters before and after correction and found that correction for background contamination systematically increased the fold change contrast for marker genes (Figure 3D). That is, correction for background contamination made marker genes more specific to the cluster they were markers of. Furthermore, additional genes were found as markers in the corrected data, that were not identified in the uncorrected data.

As a specific example, we found that correction of ambient RNA contamination changes the pattern of expression of *LYZ* in the PBMC data (Figure 3E-F). This improved the specificity of *LYZ* as a marker gene for mononuclear phagocytes (Figure 3E) by removing its expression from all other cell types, while leaving its expression in mononuclear phagocytes unchanged (Figure 3F).

Ambient RNA confounds interpretation in complex experiments

As a further test of the biological utility of our method we considered an experiment combining 7 kidney tumours processed across

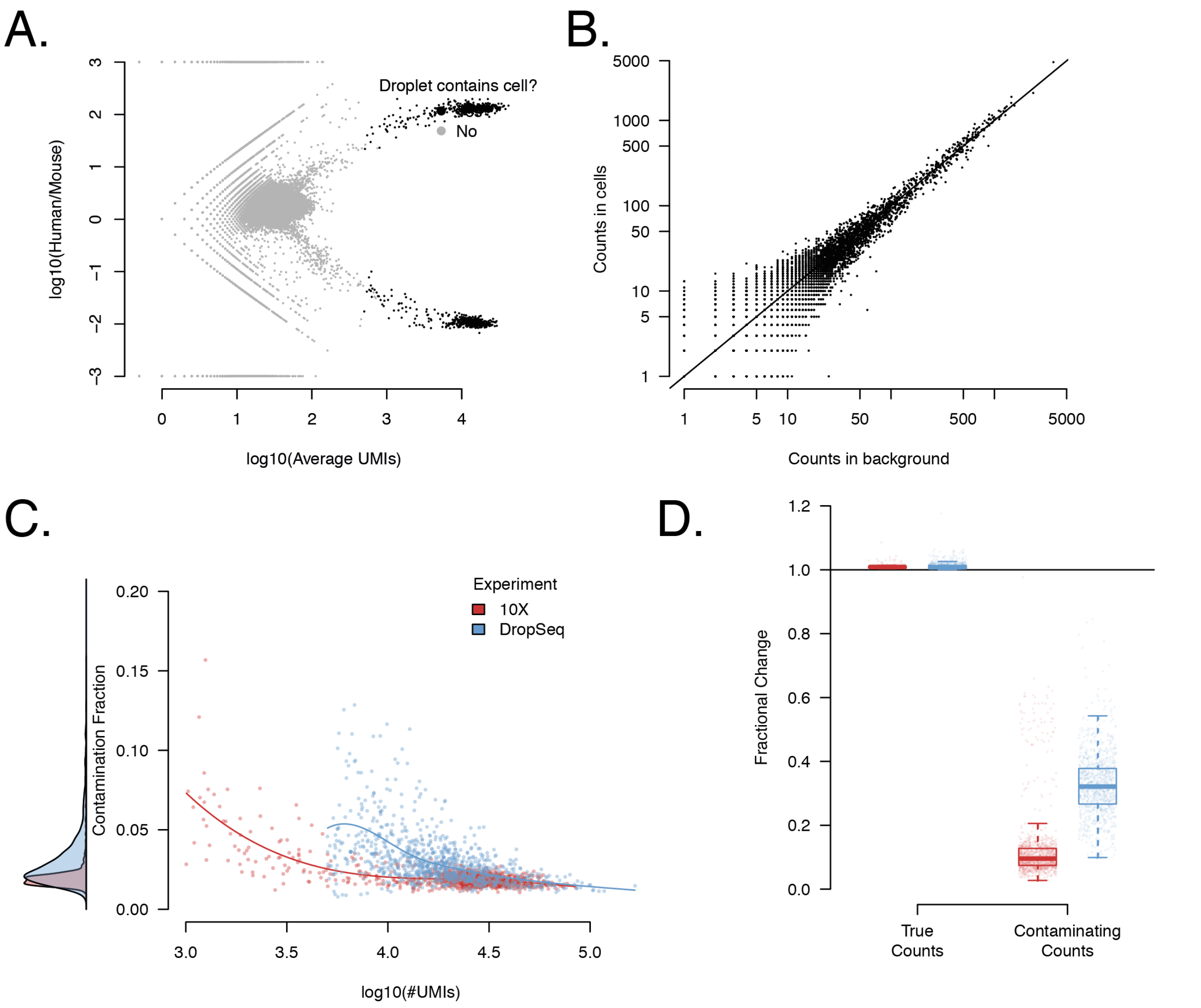

Figure 2. The properties of the cell free mRNA soup as determined using species mixing datasets. Panel **A** shows the $\log_{10}$ ratio of the number of UMIs mapping to human and mouse mRNAs for each droplet in the species mixing dataset (10X). Droplets determined to contain cells by cellranger are marked in black. Panel **B** shows the correlation of the counts in the background compared to counts averaged across cells for each gene. Counts have been sub-sampled so that the total number of counts in the background and averaged cell population is the same. Panel **C** shows the estimated contamination fraction as a function of number of UMIs in each droplet in individual cells in the species mixing dataset. Red/blue dots represent cells from the 10X/DropSeq experiments, respectively. The distribution on the left shows the marginal distribution across all cells. Panel **D** shows the fractional change in contaminating and genuine express levels after applying SoupX for the two technologies. The distribution across cells is summarised by boxplots, where the central line is the median, box boundaries are the 1st and 3rd quartiles and the whiskers extend to 1.5 times the interquartile range.

10 channels (Table S1). As with the PBMCs, we analysed corrected and uncorrected data using the Seurat package; Figure 4A shows a tSNE plot of the uncorrected data. Haemoglobin genes were used to estimate the contamination fraction in most channels (Figure S10). This choice of gene-set for estimating contamination was motivated by the ubiquitous presence of red blood cells (with red cell lysis forming part of the tissue treatment protocol) in these samples, together with the knowledge that haemoglobin genes are highly specific to red blood cells. We compared the resulting estimates of the contamination fraction with those obtained by applying the automated method and found good agreement (Figure S6).

Applying SoupX and re-analysing the kidney tumour data revealed that, in contrast to the PBMC data, many cells changed cluster and with the same clustering parameters two fewer clusters were identified in the corrected data (Figure 4B). Furthermore, we found that the expression ratio of marker genes between the cluster they mark and all other cells increased systematically after correcting for background contamination (Figure S7).

We found that the correction of background contamination changed the distribution of expression of many genes across cells in a way that would alter the biological interpretation. For example, while unlikely to be biologically misinterpreted, SoupX completely removes the expression of haemoglobin genes from all cells except red blood cells (Figure 4C).

In other cases, the misattribution of gene expression to cell types that do not truly express them could lead to false conclusions. An example of this is the cluster of T and mononuclear phagocytes (MNPs) in Figure 4A,D, which express the collagen genes *COL1A1*, *COL1A2*, and *COL3A1* before background correction. The expression of collagen genes might be interpreted as evidence that the leukocytes are

resident in the tissue. However, our method identifies that a high fraction of this expression is due to contamination (Figure 4D).

As the ambient mRNA expression profile is experiment specific, we reasoned that background contamination likely creates a batch effects. That is, two identical cells captured in different experiments will appear different due to differences in their cell free RNA composition. We therefore calculated the cross-batch entropy of the Kidney tumour data before and after background correction [20]. This analysis shows that the batch mixing entropy is increased after background correction, indicating better mixing between samples (Figure 4E).

As a further example of the biological utility of SoupX, we applied SoupX to 40 channels of human fetal liver data (Figure S8). Before correcting for background contamination, a large number of cells outside the erythroid (red blood cell) lineage express erythroid markers such as *HBB* in combination with other cell type markers. This widespread expression of multiple distinct markers could potentially indicate the presence of doublets. Application of SoupX allows this explanation to be ruled out, showing that *HBB* is only truly expressed in erythroid cell types (Figure 4F).

Application of SoupX is also able to identify those cell types where biologically unexpected combinations of genes represent genuine biology. One example of this, is the expression of the erythroid gene *GYPA* in the EI macrophage populations, which could either be the consequence of contamination or biology. Application of SoupX confirms that this expression represents genuine biology and not ambient RNA contamination (Figure S9).

Discussion

We have shown that cell free RNA is omnipresent in droplet based scRNA-seq data and have proposed a method to identify, quantify and remove its contaminating effect. We find that accounting for contamination improves the specificity of marker genes, identifies new markers, and is essential for the correct biological interpretation of complex experiments.

We have shown some potential misinterpretations of kidney tumour and fetal liver data driven by ambient mRNA contamination, but examples are sure to abound in other tissues. For instance, in endocrine tissues, it is crucial to understand which cell types secrete a particular hormone. The misassigned expression of even a single hormone gene can fundamentally change how investigators think about a cell type. Such problems will become increasingly common as efforts to compare similar cell types across tissues progress.

The best case for applying Soupx occurs when the user can specify a set of genes and cells where there is no cell endogenous expression. That is, a set of genes and cells where it is safe to assume that the only source of expression for these genes is from background contamination. The expectation is that biological knowledge of the experiment being performed will guide this choice.

For example, solid tissue experiments are frequently highly contaminated with red blood cells and red cell lysis is used to prepare the samples [15]. As such, haemoglobin genes are often ubiquitously present in the background. Furthermore, red blood cells are the only cells that produce haemoglobin under normal physiological conditions, so for the set of haemoglobin genes, it is safe to assume that there is no cell endogenous expression for cells that are not red blood cells. Finally, red blood cells express haemoglobin genes in such extreme abundance that they can be trivially identified by comparing the ratio of observed haemoglobin genes to that present in the background contamination (Figure S10)). These properties make haemoglobin genes a sensible choice for most solid tissue experiments.

Heuristics, such as the bimodal expression ranking in Figure S5, can help aid biologically motivated gene selection. However, we recognise that selecting an appropriate set of genes to estimate contamination will not always be possible. To address this issue, we include an automated contamination estimation procedure. By using all high quality marker genes identified in the data to independently estimate the contamination fraction, this method estimates the true contamination fraction by assuming inaccurate estimates of the contamination fraction are not strongly correlated (i.e., there is no preferred, incorrect estimate). We show this automation gives comparable results to the manual method.

It is also possible to manually specify the contamination fraction, which can be useful when the estimation procedures described above are deemed inaccurate or it is desirable to over-correct the data. For most applications, the consequences of manually setting an unrealistically high contamination rate are likely to be minimal. Contamination is preferentially removed from genes closest to the background expression (i.e., lowly expressed genes), meaning that setting a higher global contamination rate is unlikely to remove completely the expression of genes that are truly markers of a cell. Thus in some applications it may be preferable to over-correct for background contamination and remove a small amount of genuine signal in order to ensure that all the background contamination has been removed. We also find that our method is robust to small inaccuracies in the estimation of the global contamination rate (Figure S4).

Since SoupX was first released, several other tools have been developed that aim to remove background contamination. SoupOrCell [21] uses the identification of conflicting genotypes to identify ambient RNA contamination, limiting its application to mixed genotype experiments. Cell Bender [22] employs a deep generative model to estimate shared expression patterns likely to represent distinct cell types while simultaneously removing contamination. This deep generative model comes with a heavy computational cost compared to other tools and the output of the model (which in effect estimates $m_{g,c}$ for each cell type) provides an imputed cell profile rather than raw counts with the background "subtracted off", which SoupX provides. Finally, DecontX [23] relies on accurate clustering of the data to estimate and remove the background without the need for gene counts from empty droplets. This allows DecontX to be applied when empty droplet counts are not available, but also means the results are potentially heavily dependent on the accuracy of the clustering provided. By contrast, SoupX can be applied generally, is computationally inexpensive and does not depend heavily on accurate pre-annotation of input data.

To make our method easily applicable, we provide an R package, SoupX, which can be used to estimate and remove ambient mRNA contamination. This package is available on the comprehensive R archive network (CRAN) and is provided with a vignette to assist the user in understanding how best to apply the method. The output of the SoupX package is a corrected table of counts, which can be used as input for standard workflows and running SoupX does not add appreciably to the computational cost of standard single cell analyses. We envision background correction forming a standard part of droplet based scRNA-seq analysis pipelines.

Competing interests

The authors declare that they have no competing interests.

Author's contributions

M.D.Y. conceived the project, developed the method and wrote the manuscript. S.B. Contributed to the method development.

Acknowledgements

We thank William Heaton and Valentine Svensson for discussions about droplet sequencing; Sarah Teichmann for discussions about the methodology; Sarah Teichmann, Aaron Lun and Manasa Ramakrishna for comments and review of the manuscript and Manasa

Ramakrishna for improvements to the figures and their layout. Martin Prete for help with creating a docker version of the code. We thank Justin McManus and Mia Jaffe for discussions about all aspects of the paper, particularly around ways to automate estimation of contamination.

Data availability

The 10X species mixing dataset was the mixture of the human cell line 293T and the mouse cell line 3T3 described in [2]. We used the data mapped and quantified using Cell Ranger 1.1.0 from https://support.10xgenomics.com/single-cell-gene-expression/datasets/1.1.0/293t_3t3. The dropseq species mixing data was obtained from [14], specifically SRR1748411. The PBMC data was taken from [2]. The 'kidney tumour data set' was taken from [15]. The fetal liver data [16] is available from ArrayExpress with accession code E-MTAB-7407. The mapped data sets supporting the results of this article are available in the GigaDB repository, [To be determined].

Code availability

An implementation of the SoupX method described here can be found on github https://github.com/constantAmateur/SoupX and in CRAN https://cran.r-project.org/web/packages/SoupX/index.html. The code used to generate the results presented in this manuscript can be found here https://github.com/constantAmateur/ambientRNA_paper. A docker container, containing all code and data needed to generate the results in this paper can be obtained from https://hub.docker.com/r/constantamateur/soupxpaper.

Supplementary Materials

Notation

We refer to the observed counts in a droplet c for gene g as n_{gc}. Sums taken over a variable are represented with a ., so $n_{.c} = \sum_g n_{gc}$; the sum over all genes for droplet c. m_{gc} represents the cell endogenous counts present in a droplet c, for gene g. Similarly, o_{gc} represents the other counts in a droplet, contributed by background contamination.

We denote the fractional abundance of gene g in the soup or ambient mRNA background as b_g. This is defined such that $b_. = 1$. ρ_c represents the background contamination fraction in droplet c, defined as $o_{.c}/n_{.c}$.

We define $\mathcal{G}$ as the set of all genes that can be detected in a sequencing experiment.

Choice of count distribution

The most appropriate model for count based data, such as the counts produced by scRNA-seq is a multinomial distribution. This distribution provides the probability of observing a given partition of N counts into k genes, given the relative probabilities of each gene p_g.

In estimating how many counts to remove, where N tends to be small, we directly maximise the multinomial likelihood. However, in other cases we approximate the multinomial distribution as k Poisson distributions, which is an accurate approximation in the limit of large N and small p.

These distributions are commonly extended to include over-dispersion by using a Dirichlet multinomial or negative binomial distribution. Throughout this paper we ignore the effects of over-dispersion for computational expediency and because for most estimation procedures used the maximum likelihood estimator does not depend on the over-dispersion (e.g. estimating the mean).

Detailed description of the SoupX method

As discussed in the manuscript, SoupX aims to remove the contribution of the cell free mRNA molecules from each cell. The algorithm consists of the following three steps:

i. Estimate the ambient mRNA expression profile from empty droplets.
ii. Measure the contamination fraction, the fraction of UMIs originating from the background, in each cell.
iii. Correct the expression of each cell using the ambient mRNA expression profile and estimated contamination.

SoupX produces a modified table of counts, which can be used in place of the original count matrix in any downstream analysis tool. This supplement provides the details for each of these three parts of the SoupX method.

Background expression profiles

To calculate the expression profile of cell free mRNAs, we assume that droplets with a very low UMI count contain only cell free mRNAs. As the number of droplets with low UMI counts is very large compared to the number of cells ($\sim 10^6$ droplets versus $\sim 10^4$ cells), there is typically abundant power to accurately calculate the expression profile of the cell free background. Let $\mathcal{D}$ denote the set of all droplets with a UMI count $\alpha_l \leq n_{.c} \leq \alpha_u$. The background expression fraction for gene g, b_g, is estimated as

$$b_g = \frac{\sum_{c \in \mathcal{D}} n_{gc}}{\sum_{\tilde{g} \in \mathcal{G}} \sum_{c \in \mathcal{D}} n_{\tilde{g}c}} \tag{6}$$

That is, b_g is the fraction of counts derived from gene g in the set of empty droplets $\mathcal{D}$, normalised so that $b_. = 1$. In the species mixing data, we could directly measure the background contamination in droplets *with* cells. We used this gold standard to measure the accuracy of the estimated background as a function of the number of UMIs in the droplets used to estimate it (Figure S2). Based on this, we set $\alpha_l = 2$ and $\alpha_u = 10$ in this paper. We ignore droplets with 1 UMI to prevent errors in the droplet barcodes from contaminating our estimate of the background (although we find no evidence this is a problem for chromium 10X data). Different cut-offs may be more appropriate for different technologies, but we find good correlation between the expression profile of all droplets with less than ~ 100 counts.

Calculating the contamination fraction

SoupX needs an estimate of the global contamination fraction present in a channel. This is generally not known in advance and must be estimated from the data or provided by the user. Our method approaches this problem by trying to identify a set of gene/cell pairs for which the cell endogenous expression can be assumed to be zero. That is, the task is to identify

$$\Omega = \{c, g | m_{gc} = 0\} \tag{7}$$

For genes and cells in Ω we see from equation 2 that $n_{gc} = o_{gc}$. That is, the observed counts are purely due to background contamination.

In certain circumstances (e.g., a well annotated dataset with very specific marker genes) the set Ω may be able to be specified directly by the user. Where this is not the case, we construct Ω in a two-step procedure.

Firstly, a set of genes that are known to be very specific to a particular cell type and highly expressed in that cell type are identified. Typical examples of this are red blood cells and haemoglobin genes or immunoglobulin genes and B cells. However, this set will depend on the experiment being performed, for example the insulin

gene *INS* and pancreatic beta cells will work well in the pancreas and be useless elsewhere.

Having identified a set of genes suitable for estimating the contamination fraction, we next identify which cells definitively do not express these genes. Again, the ideal way to do this is to have a well annotated data set where this decision can be specified in a biologically motivated way. For example, when using haemoglobin genes to estimate the contamination fraction, cells annotated as red blood cells can be excluded from Ω and other cell types included.

Where this is not possible we proceed by identifying all cells for which cell endogenous expression must be non-zero. These are the cells which are significant using a Poisson p-value under the null hypothesis that the $n_{.c}$ counts are distributed in the same way as in the background distribution. That is, we identify all cells where $p_c < 0.05$ and

$$p_c = \sum_{k=n_{gc}}^{\infty} \frac{\lambda^k e^{-\lambda}}{k!} \quad (8)$$

where $\lambda = \rho_{max} b_g n_{.c}$ and ρ_{max} is the largest plausible contamination fraction (which is set to 1.0 by default). Put another way, this identifies all cells for which the fraction of counts derived from the genes of interest in the cell exceeds the fraction of counts for those genes in the background contamination.

We then cluster the data and exclude any cluster that contains a cell for which $p_c < 0.05$. This conservative approach helps ensure that the cells used to estimate the contamination are those cells with zero endogenous expression of the target genes. This approach can be made more or less conservative by adjusting the p-value threshold or clustering more or less finely.

Having constructed Ω, the set of gene/cell pairs with which to calculate the contamination fraction, we calculate the global contamination fraction for an experiment as

$$\rho = \frac{\sum_{g,c\in\Omega} n_{gc}}{\sum_{g,c\in\Omega} n_{.c} b_g} \quad (9)$$

In most cases a global estimate of ρ is sufficient and we find little evidence of large cell to cell variability in ρ. Furthermore, in most cases the counts available to estimate the contamination within each cell, $\sum_{g\in\Omega|c} n_{gc}$, is too low to provide an accurate cell level estimate.

For cases where there is a need to estimate cell specific contamination, we share information between cells using a Heirarcichal bayes model. Under the model:

$$\mu \sim \text{Normal}(0, 0.5) \quad (10)$$

$$\sigma \sim \text{Normal}(0, 1) \quad (11)$$

$$\rho \sim \text{Normal}(-4, 1) \quad (12)$$

$$\rho_c \sim \text{Normal}(\mu, \sigma) \quad (13)$$

$$n_{gc} \sim \text{Poisson}(n_{.c} b_g (\rho + \rho_c)) \forall g, c \in \Omega \quad (14)$$

That is, the data is assumed to follow a Poisson distribution, with mean given by the expected background counts times a cell-specific contamination fraction. The cell specific contamination fraction is modelled as a global contamination, plus some perturbation whose prior distribution is normally distributed, the parameters of which are determined from the data.

Automated estimation of the contamination fraction

In cases where the choice of gene set used to construct Ω and estimate ρ is not obvious, we provide an automated alternative. The intuition behind this approach is that the genes that are most often useful in estimating ρ are markers of cells in the dataset being investigated. In detail, we first cluster the data and identify highly specific marker genes of each cluster. To perform marker identification, we use an approach based on the term frequency, inverse document frequency (tf-idf) metric frequently used in natural language that has been successfully used previously [15].

Selecting marker genes for which tf-idf > 1 provides a list of genes that can be used to fed into the manual estimation procedure described above. This list is further refined by excluding any gene that is not expressed at or above the 99th expression quantile in the background profile b_g, as these genes provide the most accurate estimate of the contamination fraction. Each gene on this list is fed through the procedure above to identify Ω and estimate ρ, producing many quasi-independent estimates of ρ.

We then assume that inaccurate estimates of ρ will be randomly distributed, while true estimates will cluster around the true value. As such, the most common estimate of ρ amongst the set of estimates obtained from marker genes will represent the true value. Next we calculate the posterior probability of ρ given these estimates, utilising a gamma distribution prior with mean 0.05 and standard deviation 0.1. This broad prior has little effect on the posterior distribution, but can be set to be something more informative where prior information is available. Finally, ρ is set to the most likely value in the posterior distribution.

Correcting cell expression profiles

Having calculated the expression profile for the background b_g and the contamination fraction ρ, we use this information to modify the table of counts and remove contaminating mRNAs, m_{gc}. The obvious way to do this is by simply subtracting the contribution due to soup and setting $m_{gc} = n_{gc} - \rho n_{.c} b_g$ (or $m_{gc} = 0$ if $n_{gc} < \rho n_{.c} b_g$). Indeed, this is the maximum likelihood estimator of m_{gc} for Poisson distributed counts with mean given by equations 2 and 3.

However, following this approach will systematically undercorrect the data as the only counts for which the data will be modified is those for which $n_{gc} \geq \rho n_{.c} b_g$. To correct for this, more than $\rho n_{.c} b_g$ must be subtracted from those counts for which $n_{gc} > \rho n_{.c} b_g$. The reason for this is that the data must be modelled by a distribution that takes into account the competitive nature of sequencing, such as the multinomial distribution. That is, we need a statistical model that will find not just the most likely amount of contamination in each gene separately, but will require that the total number of counts removed from each cell must equal $\rho n_{.c}$. The usual approach of modelling the counts for each gene/cell pair with a Poisson distribution approximates a multinomial distribution (similarly the often used negative-binomial distribution is an approximation of a Dirichlet-multinomial distribution). Therefore, the true problem we want to solve is to maximise the multinomial likelihood of o_{gc} (the contaminating counts for gene g in cell c) with multinamial $n = o_{.c} = \rho n_{.c}$, and probabilities given by b_g, subject to the constraint that $0 \leq o_{gc} \leq n_{gc} \forall g, c$ (i.e., we cannot remove more counts than we observe).

We solve this problem by recognising that when gene expression "buckets" are filled in an order depending on their expression and their expression in the background. That is, as the number of counts removed increases, counts will be completely removed from genes in an order determined by $\frac{n_{gc}}{b_g}$. Knowing this, it is straightforward to calculate which genes will be completely removed for a given number of total contaminating counts and then distribute the remaining counts to be removed between all other genes proportionally to b_g.

This procedure is followed independently for each cell to produce modified counts. Where integer counts are required for downstream analysis, we round the corrected counts up to the nearest integer with probability given by the $m_{gc} - \lceil m_{gc} \rceil$.

Improving count removal using clustering

Where clustering of cells is provided, the above procedure can be improved by applying the correction procedure to counts aggregated within clusters. Doing this greatly increases the statistical power to distinguish between contamination and true expression. The value

of ρ used for cluster P is calculated as

$$\rho_P = \frac{\sum_{c \in P} n_{.c}\rho_c}{\sum_{c \in P} n_{.c}} \tag{15}$$

and the number of contaminating counts for each gene is calculated as above.

To redistribute the calculated contaminating counts to the single cell level, counts for gene g are distributed to each cell with weights given by $n_{.c}\rho_c$. This redistribution is done using the same logic as for removing counts to ensure that a cell cannot be assigned more contamination for a gene than has actually been observed.

Cells that have a higher true contamination rate than the global average will have more non-zero counts in genes with high contamination than cells with a lower contamination rate than average. Because of this, the redistribution procedure described above will assign more contaminating counts to high contamination cells and fewer to low contaminating cells, even without this information being explicitly provided. This can be seen in Figure S4, where the effective cell level contamination rate implied by correcting at the cluster level and redistributing counts is highly correlated with the true cell level contamination rate.

Processing of data sets

The DropSeq Species Mixing experiment was downloaded from the short read archive (SRR1748411) and quantified using Alevin [24] with a mixed human/mouse reference and the 'forceCells' flag set to 1 to include all barcodes.

For all 10X data sets, we used all droplets identified by cellranger as containing cells. In the species mix data, we removed any droplet with at least 1000 UMIs from *both* human and mouse genes, as these are likely doublets. For the DropSeq species mix experiment we set this threshold to 5000.

We used the Seurat package (http://satijalab.org/seurat/) to parse distinct cell types and marker genes from these preprocessed sequencing data. Raw counts of UMIs per gene in each cell were normalised using the Seurat::NormalizeData function, to implement the transformation

$$x_{gc} = \log(1 + 10^4 \hat{f}_{gc}) \tag{16}$$

where $\hat{f}_{gc}$ is the observed proportion of UMIs in droplet c from gene g; x_{gc} is the library-size normalised expression of gene g in droplet c.

Variable genes were identified using the Seurat::FindVariableGenes function with default parameters. Within Seurat, we subset the library-size normalized gene expression matrix on the variable genes, and we standardized the matrix so that the variable genes have mean 0 and standard deviation 1. We calculated the first 30 principle components of the standardized matrix, and the graph-based clustering algorithm implemented in Seurat::FindClusters evaluated the distance between cells in this 30-dimensional PCA volume. The t-SNE embedding was calculated using a perplexity of 30, and clusters were identified with the Seurat::FindClusters resolution parameter set to 1.

To identify genes specific to each cluster, we used the Seurat 'FindMarkers' function with default parameters.

These markers were then manually inspected and each cluster was assigned a cell type based on the comparison of these markers to the literature (particularly [25, 26, 27, 15]).

For the foetal liver data, we used pre-supplied UMAP coordinates and cell labels, generated as previously described[16]. Contamination fractions were determined independently for each channel using the automated method, except for those channels where this could not return a result due to too few cells, in which case we manually set the contamination to 10%, which was roughly the average of all other channels.

References

1. Zilionis R, Nainys J, Veres A, Savova V, Zemmour D, Klein AM, et al. Single-cell barcoding and sequencing using droplet microfluidics. Nature Protocols 2016 Dec;12(1):44–73.
2. Zheng GXY, Terry JM, Belgrader P, Ryvkin P, Bent ZW, Wilson R, et al. Massively parallel digital transcriptional profiling of single cells. Nature communications 2017 Jan;8:14049.
3. Hashimoto S, Tabuchi Y, Yurino H, Hirohashi Y, Deshimaru S, Asano T, et al. Comprehensive single-cell transcriptome analysis reveals heterogeneity in endometrioid adenocarcinoma tissues. Scientific reports 2017 Oct;7(1):14225.
4. Bach K, Pensa S, Grzelak M, Hadfield J, Adams DJ, Marioni JC, et al. Differentiation dynamics of mammary epithelial cells revealed by single-cell RNA sequencing. Nature communications 2017 Dec;8(1):2128.
5. Daniszewski M, Senabouth A, Nguyen QH, Crombie DE, Lukowski SW, Kulkarni T, et al. Single cell RNA sequencing of stem cell-derived retinal ganglion cells. Scientific data 2018 Feb;5:180013.
6. Stephenson W, Donlin LT, Butler A, Rozo C, Bracken B, Rashidfarrokhi A, et al. Single-cell RNA-seq of rheumatoid arthritis synovial tissue using low-cost microfluidic instrumentation. Nature communications 2018 Feb;9(1):791.
7. Chen YJJ, Friedman BA, Ha C, Durinck S, Liu J, Rubenstein JL, et al. Single-cell RNA sequencing identifies distinct mouse medial ganglionic eminence cell types. Scientific reports 2017 Mar;7:45656.
8. Alberti-Servera L, von Muenchow L, Tsapogas P, Capoferri G, Eschbach K, Beisel C, et al. Single-cell RNA sequencing reveals developmental heterogeneity among early lymphoid progenitors. The EMBO journal 2017 Dec;36(24):3619–3633.
9. Regev A, Teichmann SA, Lander ES, Amit I, Benoist C, Birney E, et al. The Human Cell Atlas. eLife 2017 Dec;6:503.
10. Rozenblatt-Rosen O, Stubbington MJT, Regev A, Teichmann SA. The Human Cell Atlas: from vision to reality. Nature 2017 Oct;550(7677):451–453.
11. Gayoso A, Shor J, Brand R. DoubletDetection: Identifying Technical Error in Single-cell RNA-sequencing Data 2017;https://github.com/JonathanShor/DoubletDetection/blob/master/docs/DoubletDetection.pdf, https://github.com/JonathanShor/DoubletDetection/blob/master/docs/DoubletDetection.pdf.
12. Ilicic T, Kim JK, Kolodziejczyk AA, Bagger FO, McCarthy DJ, Marioni JC, et al. Classification of low quality cells from single-cell RNA-seq data. Genome biology 2016 Feb;17(1):29.
13. Wolock SL, Lopez R, Klein AM. Scrublet: Computational Identification of Cell Doublets in Single-Cell Transcriptomic Data. Cell Syst 2019 Apr;8(4):281–291.
14. Macosko EZ, Basu A, Satija R, Nemesh J, Shekhar K, Goldman M, et al. Highly Parallel Genome-wide Expression Profiling of Individual Cells Using Nanoliter Droplets. Cell 2015 May;161(5):1202–1214.
15. Young MD, Mitchell TJ, Vieira Braga FA, Tran MGB, Stewart BJ, Ferdinand JR, et al. Single-cell transcriptomes from human kidneys reveal the cellular identity of renal tumors. Science 2018;361(6402):594–599. http://science.sciencemag.org/content/361/6402/594.
16. Popescu DM, Botting RA, Stephenson E, Green K, Webb S, Jardine L, et al. Decoding human fetal liver haematopoiesis. Nature 2019 10;574(7778):365–371.
17. Svensson V, Natarajan KN, Ly LH, Miragaia RJ, Labalette C, Macaulay IC, et al. Power analysis of single-cell RNA-sequencing experiments. Nat Methods 2017 Apr;14(4):381–387.

18. Satija R, Farrell JA, Gennert D, Schier AF, Regev A. Spatial reconstruction of single-cell gene expression data. Nat Biotechnol 2015 May;33(5):495–502.
19. Butler A, Satija R. Integrated analysis of single cell transcriptomic data across conditions, technologies, and species. bioRxiv 2017;p. 164889.
20. Haghverdi L, Lun ATL, Morgan MD, Marioni JC. Batch effects in single-cell RNA-sequencing data are corrected by matching mutual nearest neighbors. Nat Biotechnol 2018 06;36(5):421–427.
21. Heaton H, Talman AM, Knights A, Imaz M, Gaffney D, Durbin R, et al. souporcell: Robust clustering of single cell RNAseq by genotype and ambient RNA inference without reference genotypes. bioRxiv 2019;https://www.biorxiv.org/content/early/2019/09/10/699637.
22. Fleming SJ, Marioni JC, Babadi M. CellBender remove-background: a deep generative model for unsupervised removal of background noise from scRNA-seq datasets. bioRxiv 2019;https://www.biorxiv.org/content/early/2019/10/03/791699.
23. Yang S, Corbett SE, Koga Y, Wang Z, Johnson WE, Yajima M, et al. Decontamination of ambient RNA in single-cell RNA-seq with DecontX. Genome Biol 2020 03;21(1):57.
24. Srivastava A, Malik L, Smith T, Sudbery I, Patro R. Alevin efficiently estimates accurate gene abundances from dscRNA-seq data. Genome biology 2019;20(1):65.
25. Chabardès-Garonne D, Mejéan A, Aude JC, Cheval L, Di Stefano A, Gaillard MC, et al. A panoramic view of gene expression in the human kidney. Proceedings of the National Academy of Sciences of the United States of America 2003 Nov;100(23):13710–13715.
26. Habuka M, Fagerberg L, Hallström BM, Kampf C, Edlund K, Sivertsson Å, et al. The kidney transcriptome and proteome defined by transcriptomics and antibody-based profiling. PLoS One 2014;9(12):e116125.
27. Lee JW, Chou CL, Knepper MA. Deep Sequencing in Microdissected Renal Tubules Identifies Nephron Segment-Specific Transcriptomes. Journal of the American Society of Nephrology : JASN 2015 Nov;26(11):2669–2677.

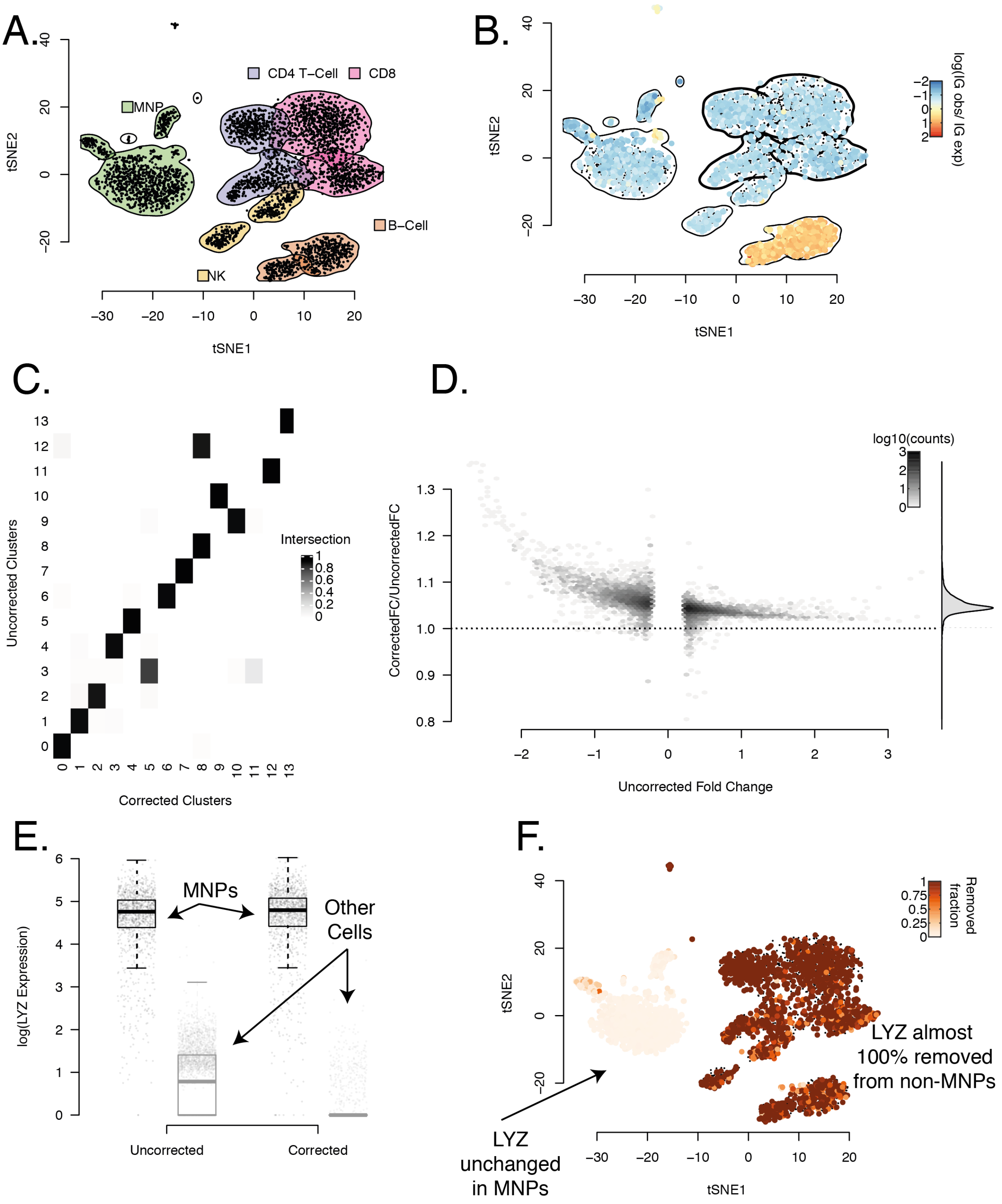

Figure 3. The PBMC dataset and how it changes when background correction is applied. Panel **A** shows a tSNE representation of the data, with cluster boundaries shown by density contours and shaded according to the cell type they represent. Abbreviations: MNP = nononuclear phagocytes, NK = natural killer cells. Panel **B** uses the same tSNE representation, but cells are now coloured by their rate of expression of immunoglobulin genes compared to the rate at which immunoglobulin is expressed in the background on a $\log_{10}$ scale. Positive values correspond to higher immunoglobulin expression in a cell than in the background with values above significantly above 0 only possible if the cell endogenously expresses immunoglobulin. The density contours of the clusters with no cell that endogenously expresses immunoglobulin (as determined by a Poisson test) are marked in bold and used to estimate the global contamination ratio. Panel **C** shows the fraction of cells shared between clusters determined with the same parameters before and after application of SoupX. Panel **D** shows the improvement in marker specificity following application of SoupX. All genes that are markers of a cluster either before or after correction are identified and their expression log fold change relative to the clusters they do not mark is calculated before and after correction. The y-axis of this plot shows the fractional change in log fold change for after applying SoupX for all genes. Genes are grouped into bins for ease of representation, with the number of genes in each bin given by the colour scale. The marginal distribution across all genes is shown on the right and the dotted line corresponds to no change in marker specificity after correction. Panel **E** illustrates the improvement in marker sensitivity for the gene *LYZ*, which is a marker for mononuclear phagocytes. The corrected and uncorrected expression levels are shown split by cells labelled as mononuclear phagocytes (MNPs) and all others. Panel **F** shows this same change in expression on the tSNE map, where the colour scale represents the fraction of *LYZ* expression that has been removed by SoupX.

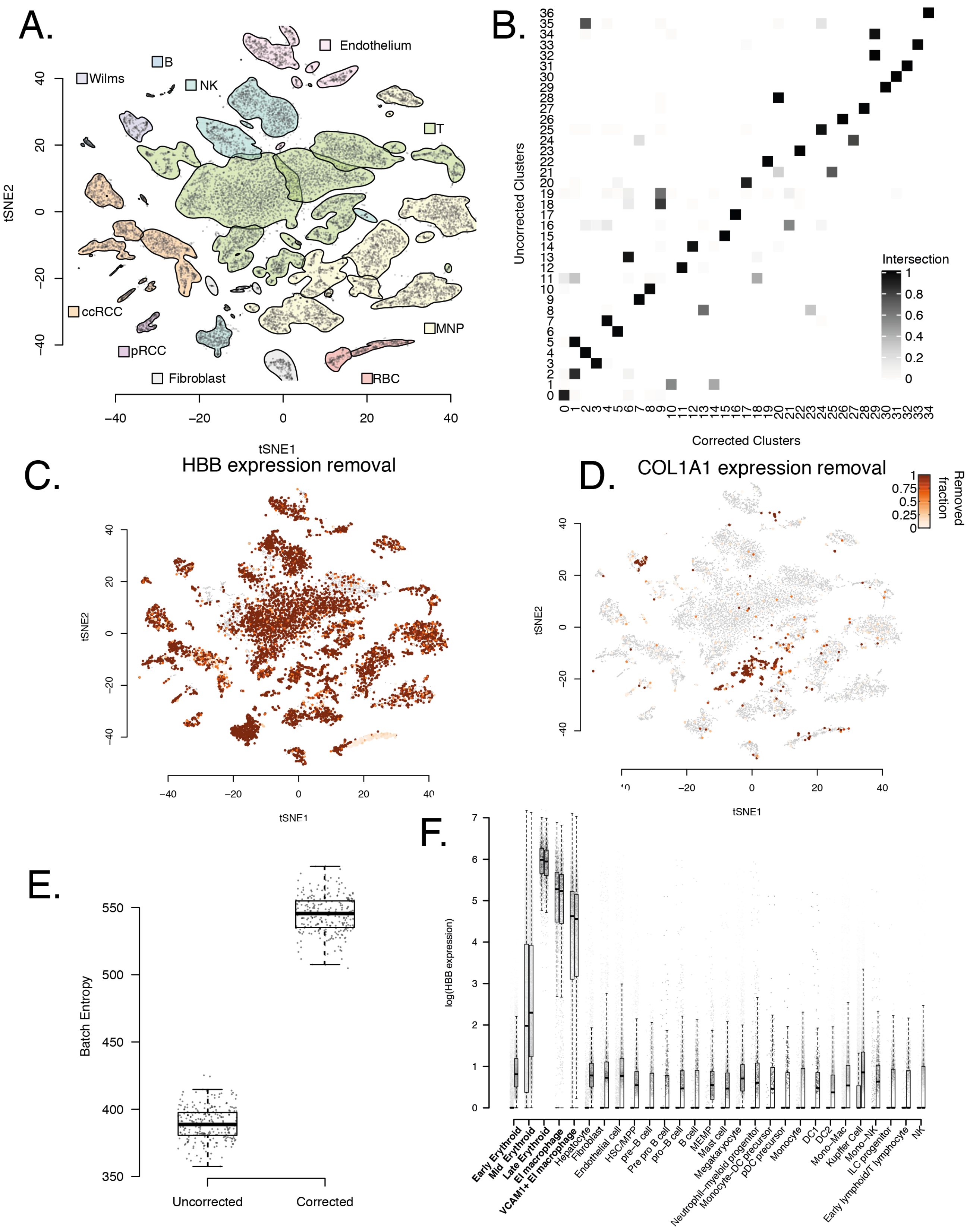

Figure 4. The application of SoupX to complex, multi-channel data. Panel **A** shows a tSNE representation of the data, with cluster boundaries shown by density contours and shaded according to the cell type they represent. Abbreviations: ccRCC = clear cell renal cell carcinoma cells, pRCC papillary cell renal cell carcinoma, RBC = red blood cells, MNP = mononuclear phagocytes. Panel **B** shows the fraction of cells shared between clusters determined with the same parameters before and after application of SoupX. Panel **C** illustrates the improvement in marker sensitivity for the gene *HBB*, which is a marker for red blood cells. The colour scale represents the fraction of *HBB* expression that has been removed by SoupX. Panel **D** the same as **C** but for *COL1A1*. Panel **E** shows the cross batch entropy before and after SoupX has been applied. The entropy measures the level of local mixing (100 nearest neighbours) for 100 cells selected from each cluster [20]. Panel **F** shows the distribution of *HBB* expression (y-axis, log scale) in the fetal liver data by cell type (x-axis), with the erythroid lineage marked in bold. For each cell type, the expression distribution is shown before (right) and after (left) application of SoupX. Dots represent individual cells and box plots show the distribution of expression values.

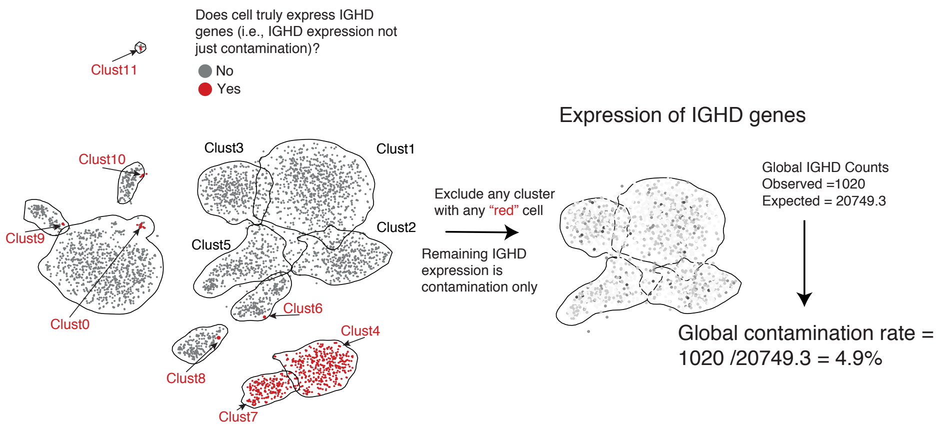

Figure S1. Schematic illustrating the procedure used to estimate the global contamination rate using the gene *IGHD* on the PBMC data. On the left, individual cells are marked red when their expression of *IGHD* is higher than would be possible even if the cell were nothing but contamination. That is, cells where *IGHD* must be endogenously expressed are marked red. Any cluster containing such a cell is excluded, and the global contamination fraction is estimated using cells in the remaining clusters (right of plot).

**Dataset**	**Name**	**Platform**	**Num Channels**	**Sample type**	**Source**
SpeciesMix	10X	10X	1	Human and Mouse cell lines	[2]
SpeciesMix	DropSeq	DropSeq	1	Human and Mouse cell lines	[14]
PBMC	PBMC	10X	1	Peripheral blood mononuclear cells	[2]
KidneyTumour	Wilms1	10X	6	Wilms' tumour	[15]
KidneyTumour	Wilms2	10X	3	Wilms' tumour	[15]
KidneyTumour	Wilms3	10X	3	Wilms' tumour	[15]
KidneyTumour	PapRCC	10X	2	Papillary cell renal cell carcinoma	[15]
KidneyTumour	RCC1	10X	4	Clear cell renal cell carcinoma	[15]
KidneyTumour	RCC2	10X	4	Clear cell renal cell carcinoma	[15]
KidneyTumour	VHL_RCC	10X	2	Clear cell renal cell carcinoma	[15]
FetalLiver	FetalLiver	10X	40	Human fetal liver	[16]

Table S1. Sample information for the different datasets used in this paper.

**Dataset**	**Sample**	**Correlation**
SpeciesMix	10X	0.94
SpeciesMix	DropSeq	0.92
PBMC	PBMC	0.96
KidneyTumour	Wilms3_Kid_T_ldc_1_1	0.92
KidneyTumour	Wilms3_Kid_T_ldc_1_2	0.93
KidneyTumour	Wilms3_Kid_T_ldc_1_3	0.93
KidneyTumour	Wilms2_Kid_T_ldc_1_1	0.73
KidneyTumour	Wilms2_Kid_T_ldc_1_2	0.73
KidneyTumour	Wilms2_Kid_T_ldc_1_3	0.77
KidneyTumour	Wilms1_Kid_R_ldc_1_1	0.75
KidneyTumour	Wilms1_Kid_R_ldc_1_2	0.74
KidneyTumour	Wilms1_Kid_R_ldc_1_3	0.85
KidneyTumour	Wilms1_Kid_T_ldc_1_1	0.84
KidneyTumour	Wilms1_Kid_T_ldc_1_2	0.86
KidneyTumour	Wilms1_Kid_T_ldc_1_3	0.84
KidneyTumour	VHL_Kid_T_ldc_1_1	0.82
KidneyTumour	VHL_Kid_T_ldc_1_2	0.81
KidneyTumour	RCC2_Kid_T_ldc_1_1	0.81
KidneyTumour	RCC2_Kid_T_ldc_1_2	0.84
KidneyTumour	RCC2_Kid_T_ldc_2_1	0.89
KidneyTumour	RCC2_Kid_T_ldc_2_2	0.89
KidneyTumour	RCC1_Kid_T_ldc_1_1	0.86
KidneyTumour	RCC1_Kid_T_ldc_1_2	0.84
KidneyTumour	RCC1_Kid_T_ldc_2_1	0.87
KidneyTumour	RCC1_Kid_T_ldc_2_2	0.87
KidneyTumour	pRCC_Kid_T_ldc_1_1	0.94
KidneyTumour	pRCC_Kid_T_ldc_1_2	0.94

Table S2. Pearson correlation coefficient between the background contamination profile and all cells in a channel averaged, after removing the genes above the 99th expression quantile.

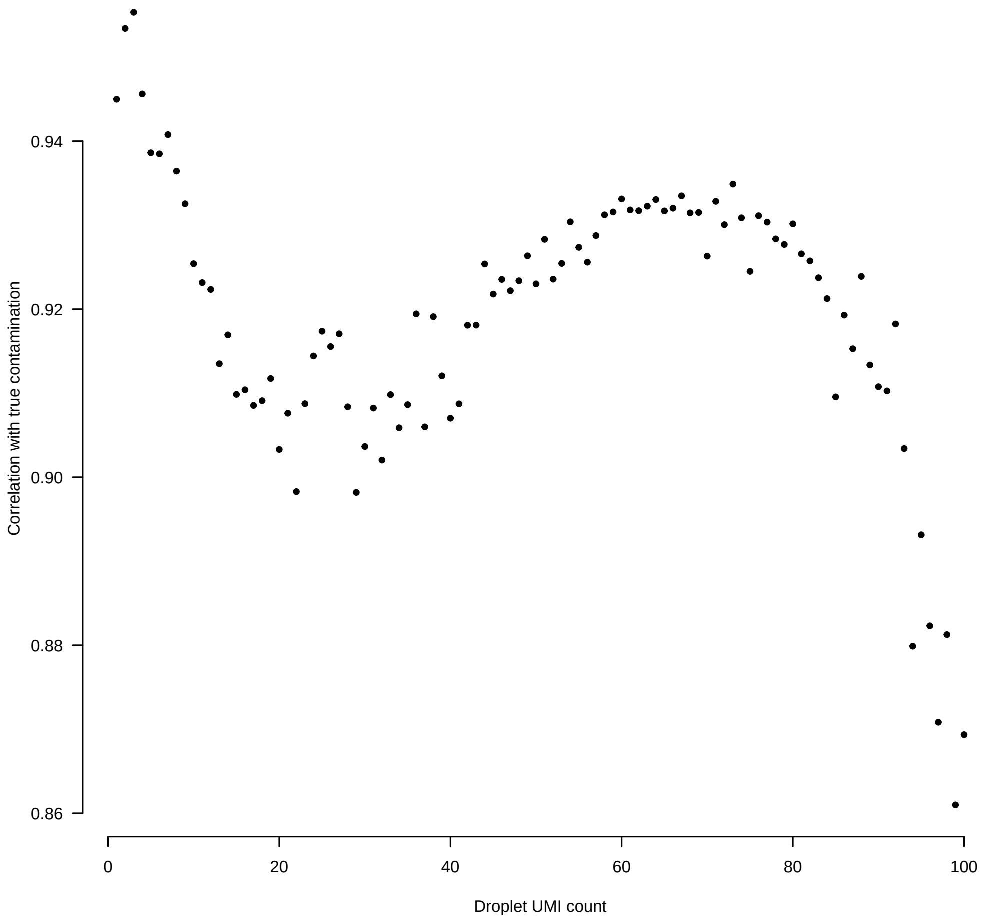

Figure S2. The correlation between "true background", which is defined by aggregating across mouse transcripts in human cells and visa versa, with the background expression profile derived using only droplets with the total number of UMIs given on the x-axis.

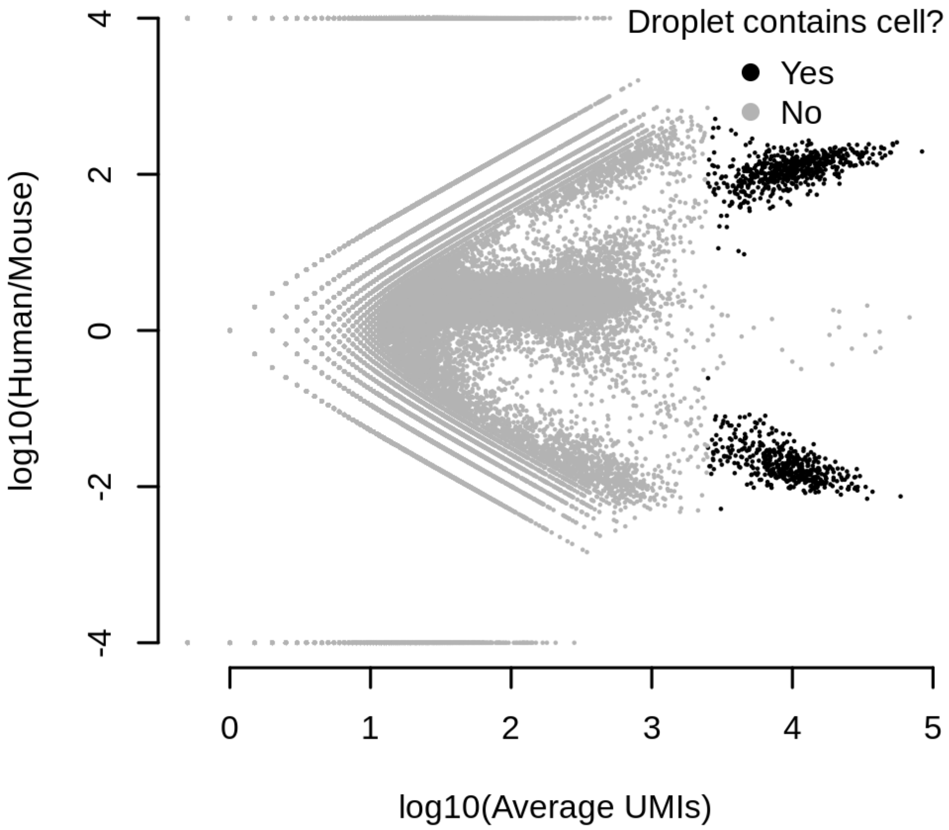

Figure S3. The ratio of human to mouse transcripts on a $\log_{10}$ scale (y-axis) for all droplets in the dropseq species mixing experiment. Droplets containing cells are marked in black. The x-axis gives the average number of UMIs between human and mouse for each cell.

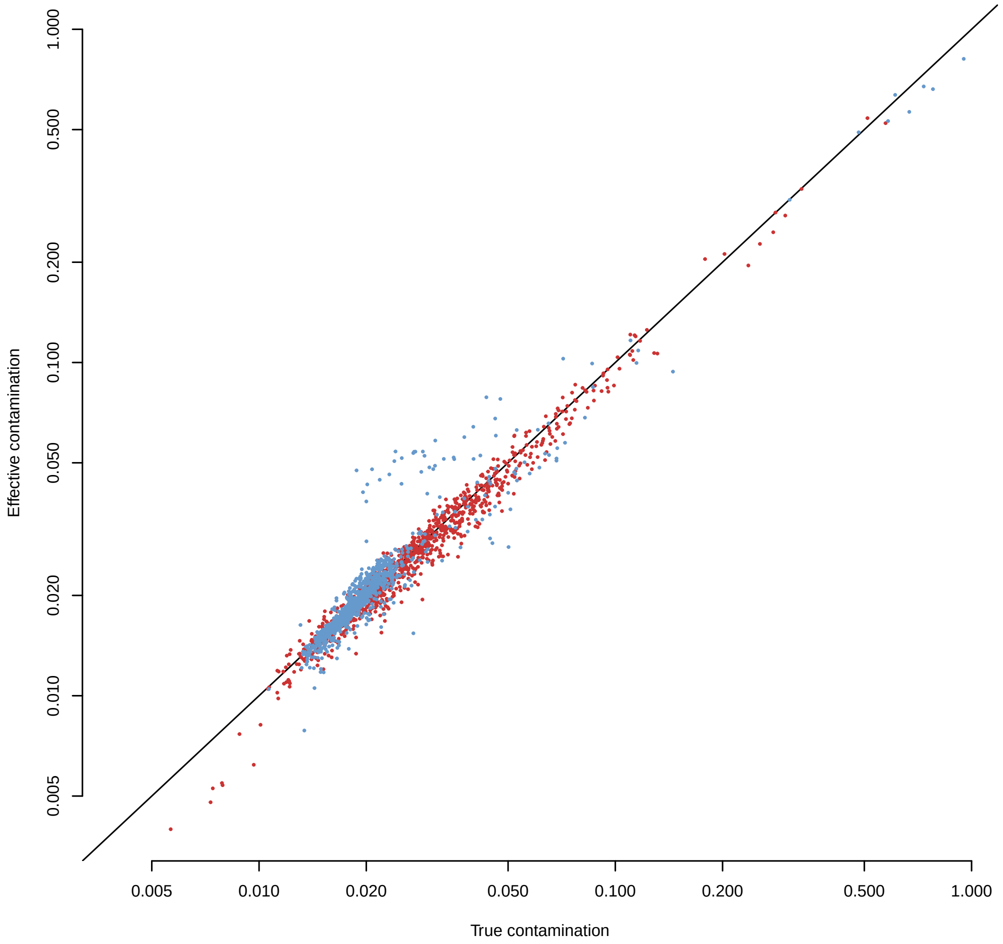

Figure S4. The x-axis gives the true contamination rate measured using the cross-species transcripts in each cell. The y-axis gives the effective contamination rate obtained by applying SoupX at the cluster level using a constant global contamination rate, calculated as the fraction of removed counts by the application of SoupX. The line shows perfect correlation and red/blue dots represent the 10X/DropSeq speciesmix experiments respectively.

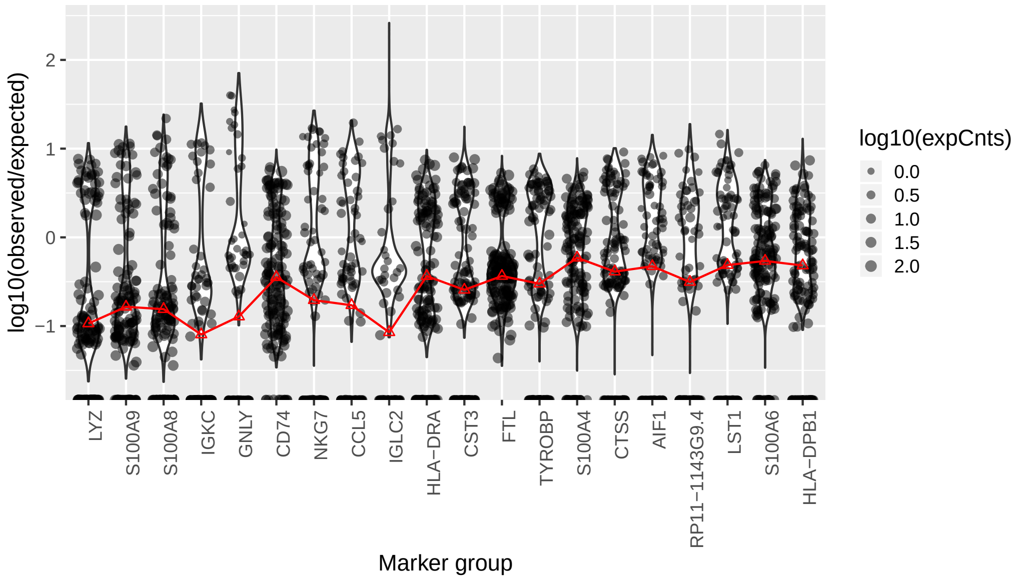

Figure S5. Distribution of expression relative to background for genes in the PBMC data. The red line indicates the global estimate of the contamination fraction that would be obtained if just that gene were used to estimate contamination. Genes which are most useful for contamination estimation have a bimodal distribution, with cells genuinely expressing the gene yielding a value on the y-axis greater than 0 and cells that do not express the gene having a value clustered around the true contamination rate.

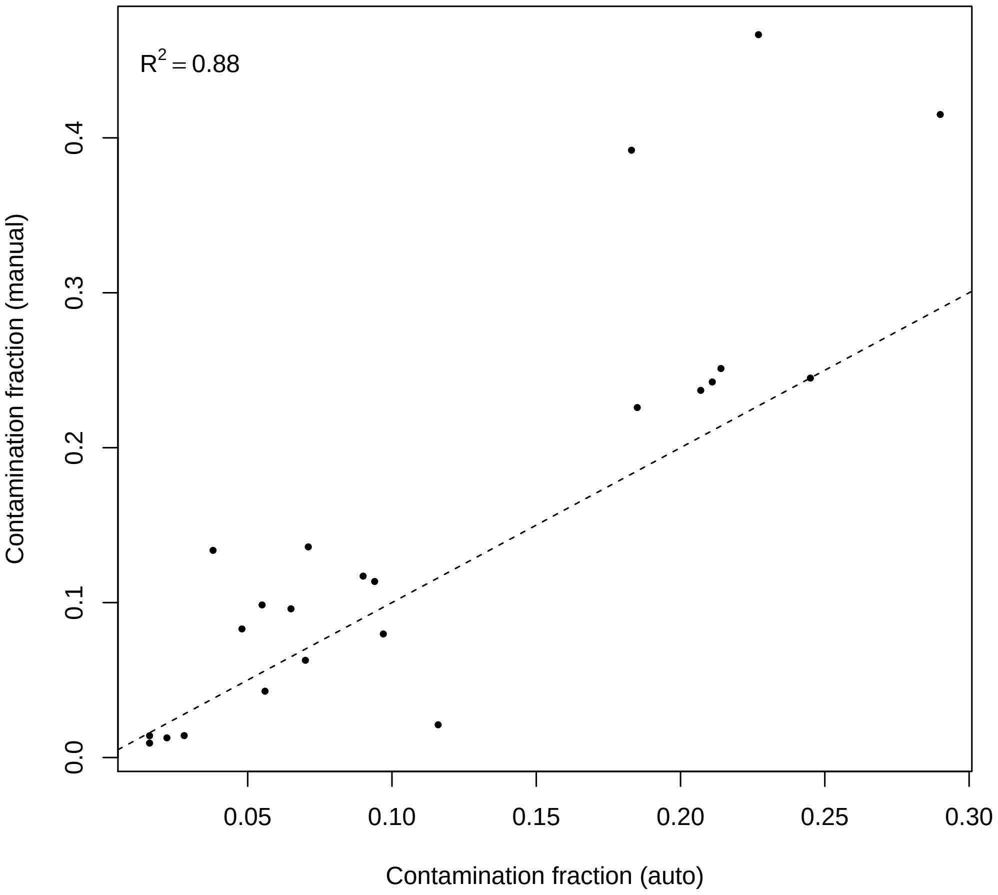

Figure S6. Comparison of the contamination fraction estimated by the automated method (x-axis) and by manually supplying a gene set (y-axis), for each channel in the kidney tumour data. The dashed line indicates perfect correlation and the Pearson's correlation is shown in the top-left.

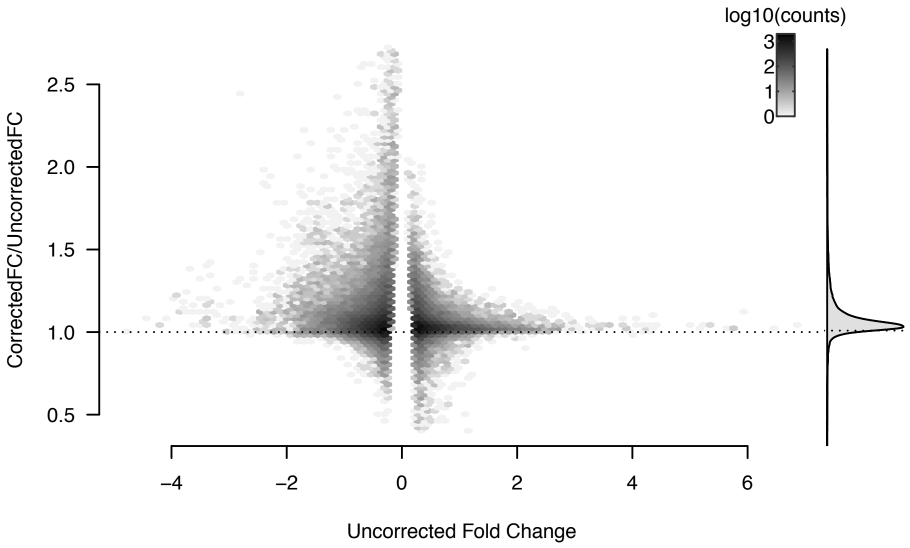

Figure S7. The improvement in marker specificity following application of SoupX to the kidney tumour data. Note the different scale of the y-axis compared to Figure 3. All genes that are markers of a cluster either before or after correction are identified and their expression log fold change relative to the clusters they do not mark is calculated before and after correction. The y-axis of this plot shows the fractional change in logFC for after applying SoupX for all genes. Genes are grouped into bins for ease of representation, with the number of genes in each bin given by the colour scale. The marginal distribution across all genes is shown on the right and the dotted line corresponds to no change in marker specificity after correction.

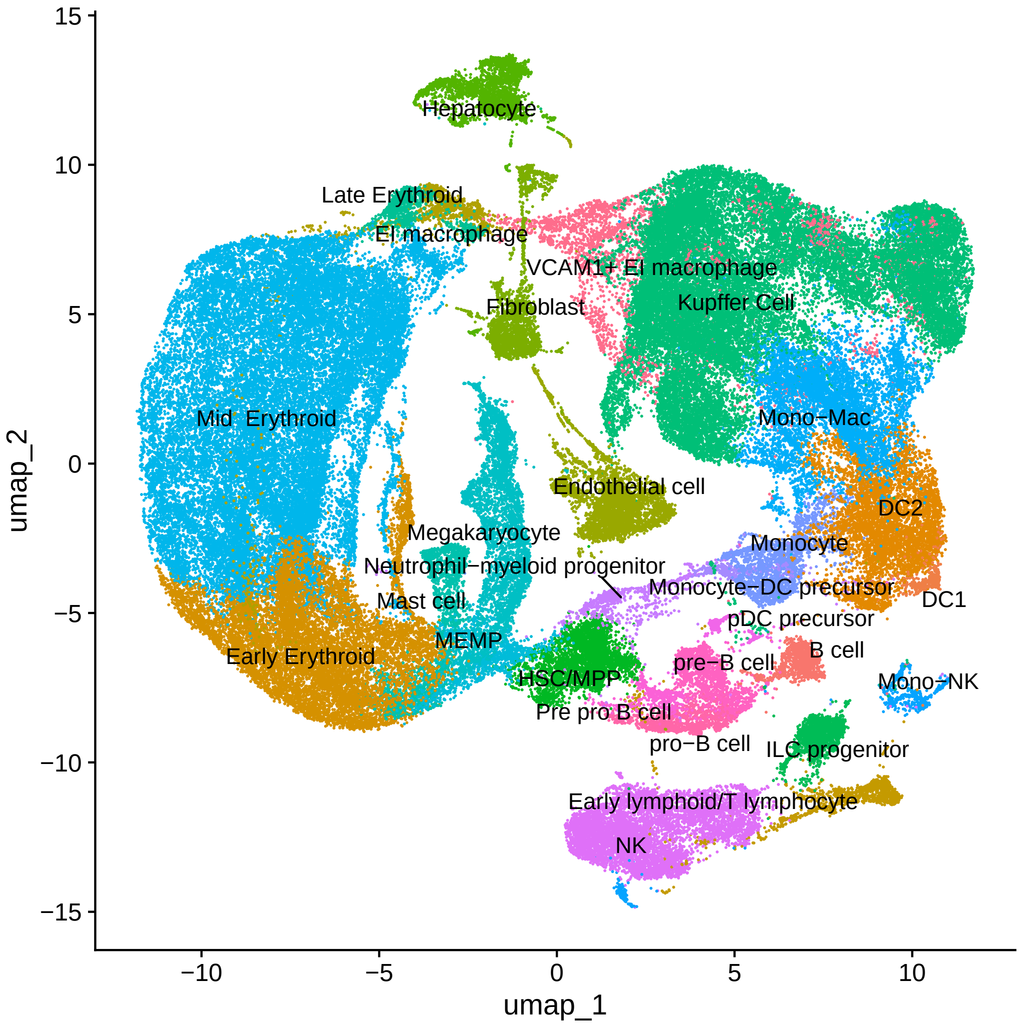

Figure S8. Uniform manifold approximation and projection (UMAP) representation of the single cell fetal data. Each point is coloured by its cell type and a cell type label is placed at the position of the average cell.

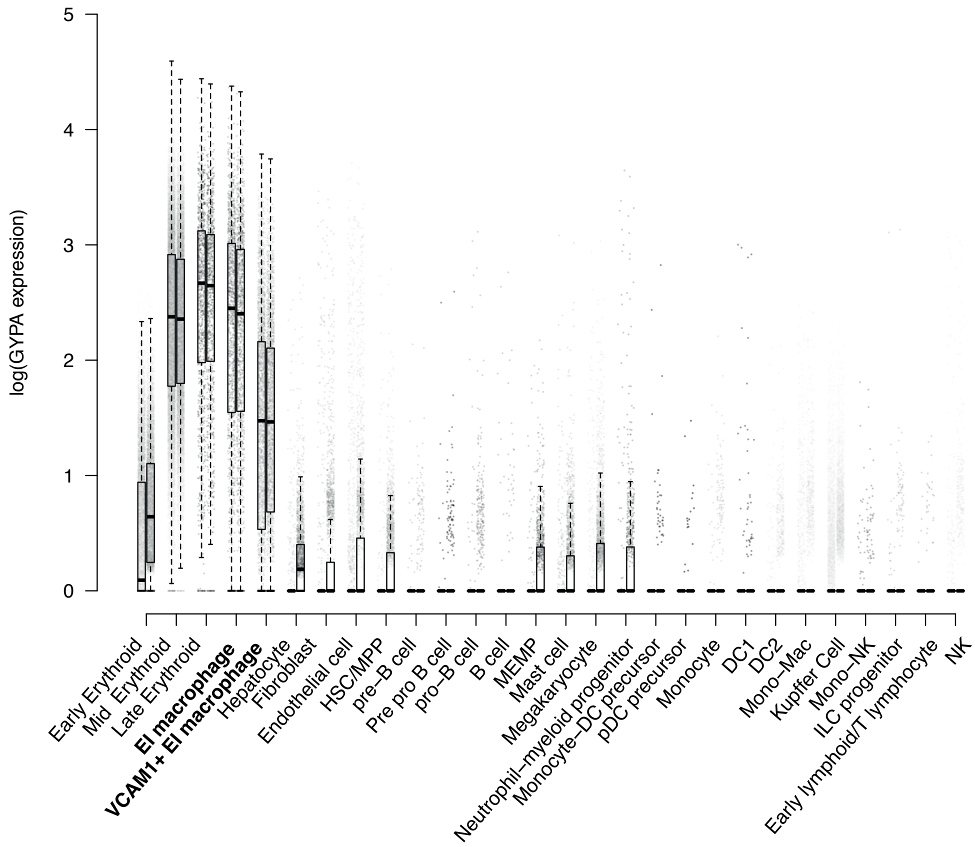

Figure S9. Normalised gene expression of *GYPA* (y-axis) in fetal liver data by cell type before and after ambient RNA removal by SoupX (x-axis). The cell types on the x-axis represent the different cell types as annotation in Figure S8. For each cell type, boxplots indicate the median, quartiles and 1.5 times the inter-quartile range for cells after SoupX correction (left) and before (right). For each distribution, each cell's expression is also shown with horizontal jitter and transparency inversely proportional to the number of cells of that type. The two EI Macrophage populations are emphasised in bold.

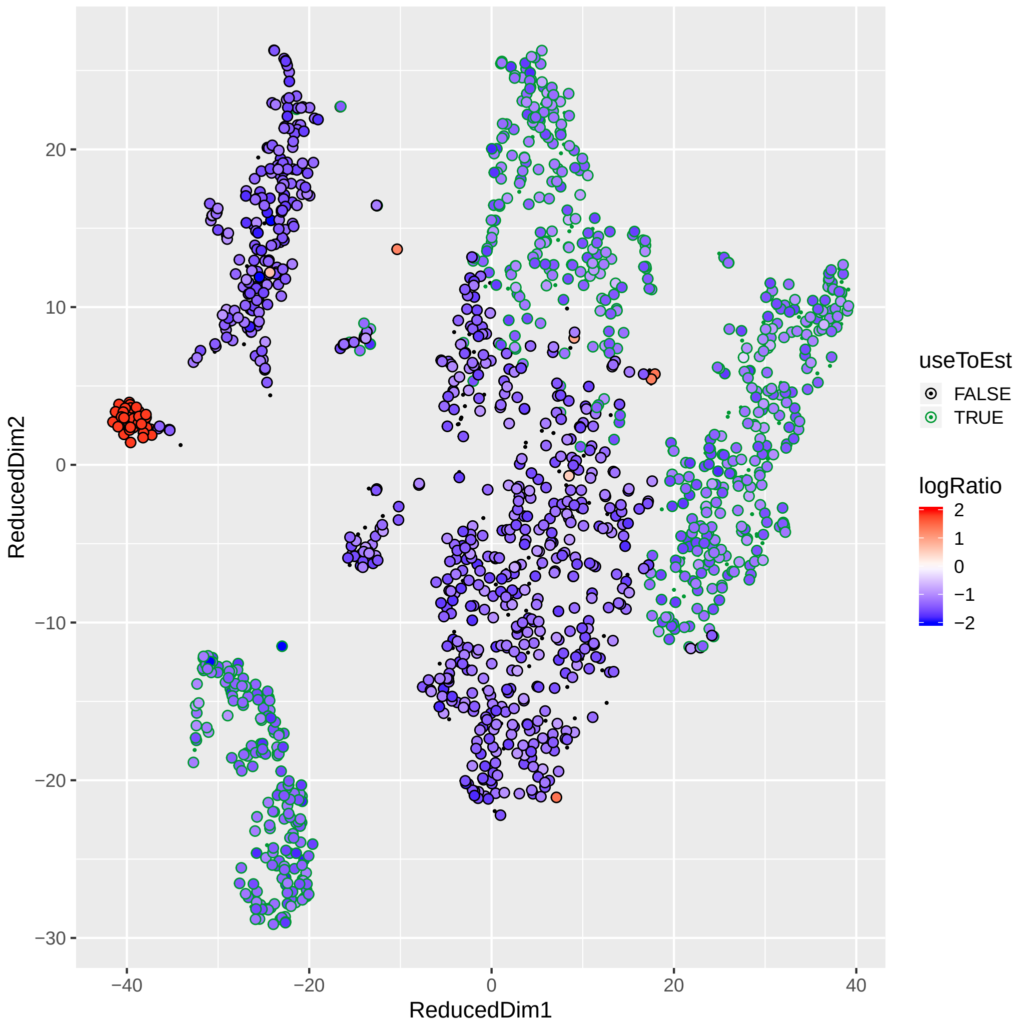

Figure S10. The fractional expression of haemoglobin genes in each cell, relative to the rate of expression in the background in one of the kidney tumour channels. This fraction is given by the colour of each point on a log scale. Points that have been determined to not endogenously express haemoglobin genes are marked with a green outline. The x and y axis are the tSNE coordinates supplied by cellranger for this channel.

Click here to access/download
Supplementary Material
ResponseToReviewersRound2.docx
