# Supplementary material for: SoupX removes ambient RNA contamination from droplet-based single-cell RNA sequencing data
Source: Gigascience. 2020 Dec 26;9(12):giaa151. doi: 10.1093/gigascience/giaa151 (PMC7763177; doi:10.1093/gigascience/giaa151)
Supplement: giaa151_Supplemental_Figures_and_Tables [file giaa151_supplemental_figures_and_tables.zip › FigureS1.pdf]

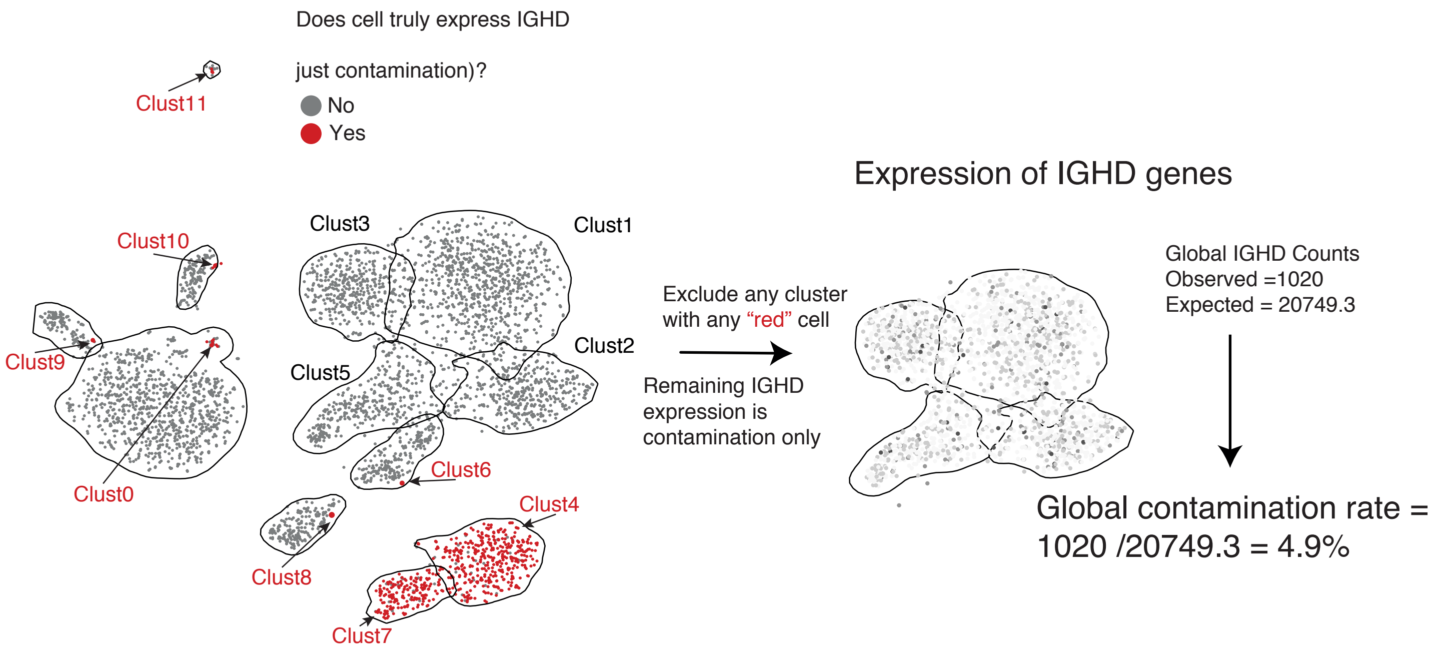

Supplementary Figure S1. Schematic illustrating the procedure used to estimate the global contamination rate using the gene IGHD on the PBMC data. On the left, individual cells are marked red when their expression of IGHD is higher than would be possible even if the cell were nothing but contamination. That is, cells where IGHD must be endogenously expressed are marked red. Any cluster containing such a cell is excluded, and the global contamination fraction is estimated using cells in the remaining clusters (right of plot).
