# Supplementary material for: SoupX removes ambient RNA contamination from droplet-based single-cell RNA sequencing data
Source: Gigascience. 2020 Dec 26;9(12):giaa151. doi: 10.1093/gigascience/giaa151 (PMC7763177; doi:10.1093/gigascience/giaa151)
Supplement: giaa151_Supplemental_Figures_and_Tables [file giaa151_supplemental_figures_and_tables.zip › FigureS10.pdf]

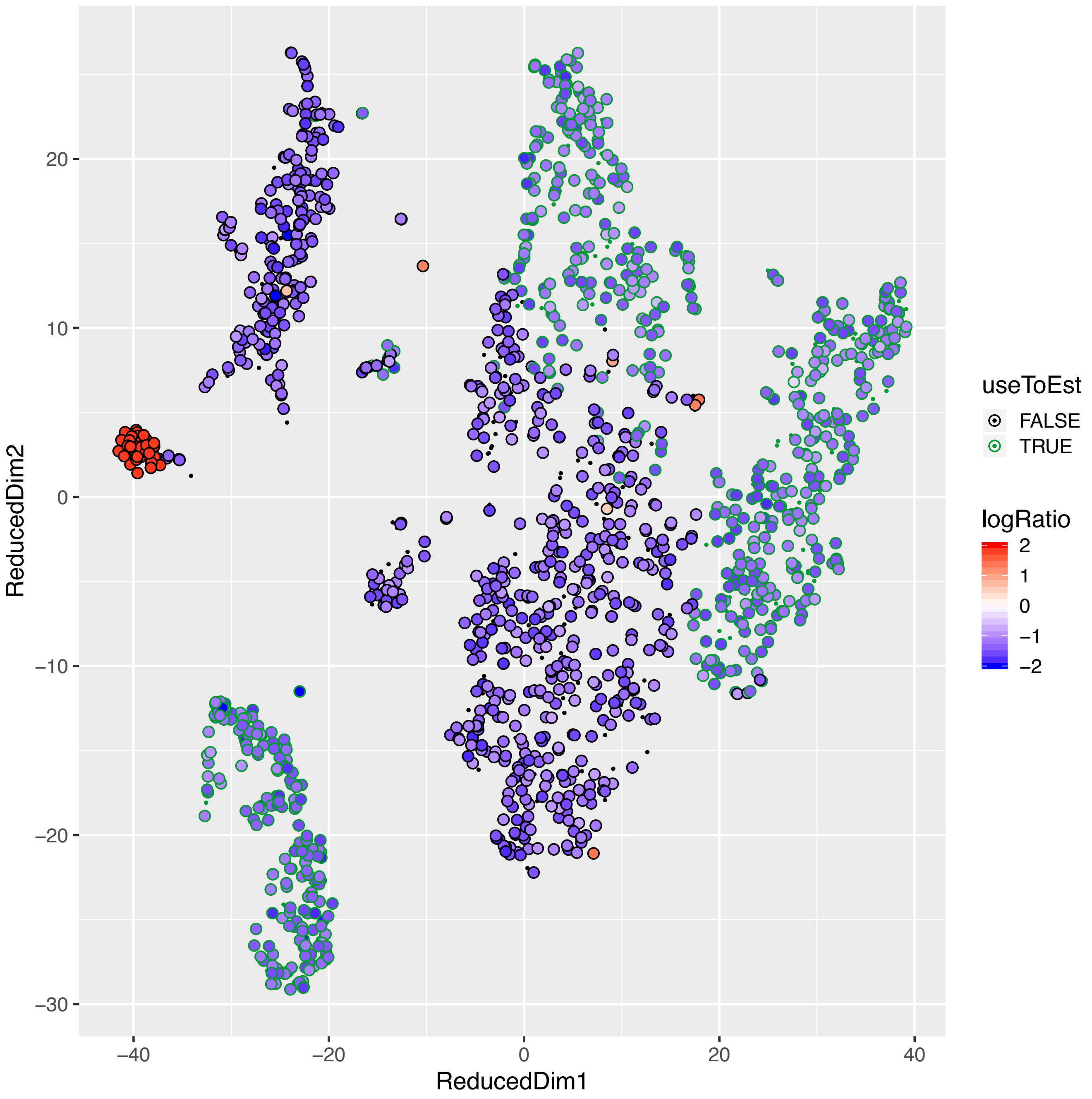

Supplementary Figure S10. The fractional expression of haemoglobin genes in each cell, relative to the rate of expression in the background in 1 of the kidney tumour channels. This fraction is given by the colour of each point on a log scale. Points that have been determined to not endogenously express haemoglobin genes are marked with a green outline. The x- and y-axis are the tSNE coordinates supplied by cellranger for this channel.
