# Supplementary material for: SoupX removes ambient RNA contamination from droplet-based single-cell RNA sequencing data
Source: Gigascience. 2020 Dec 26;9(12):giaa151. doi: 10.1093/gigascience/giaa151 (PMC7763177; doi:10.1093/gigascience/giaa151)
Supplement: giaa151_Supplemental_Figures_and_Tables [file giaa151_supplemental_figures_and_tables.zip › FigureS2.pdf]

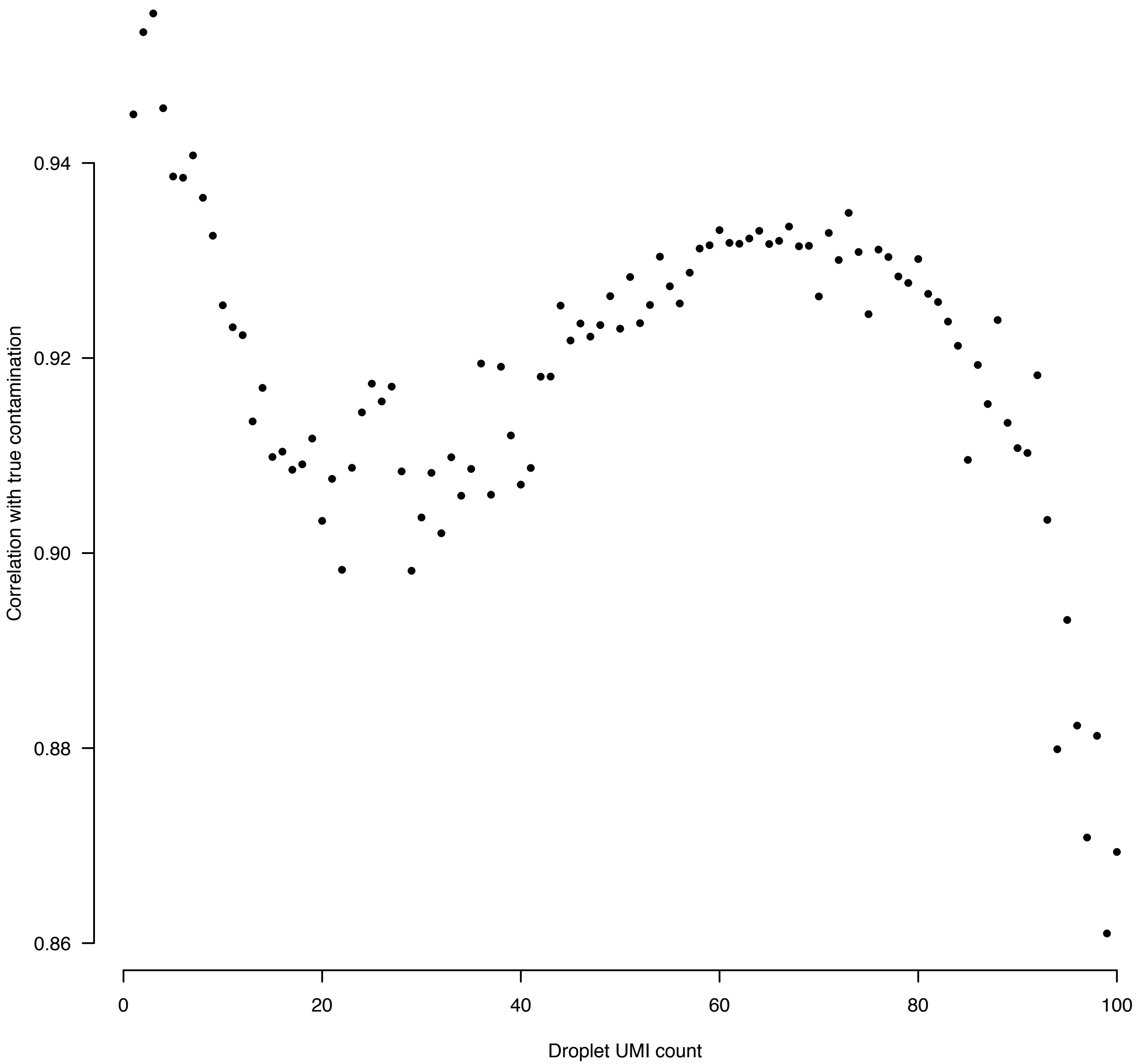

Supplementary Figure S2. The correlation between ”true background,” which is defined by aggregating across mouse transcripts in human cells and vice versa, with the background expression profile derived using only droplets. Total number of UMIs is given on the x-axis.
