# Supplementary material for: SoupX removes ambient RNA contamination from droplet-based single-cell RNA sequencing data
Source: Gigascience. 2020 Dec 26;9(12):giaa151. doi: 10.1093/gigascience/giaa151 (PMC7763177; doi:10.1093/gigascience/giaa151)
Supplement: giaa151_Supplemental_Figures_and_Tables [file giaa151_supplemental_figures_and_tables.zip › FigureS3.pdf]

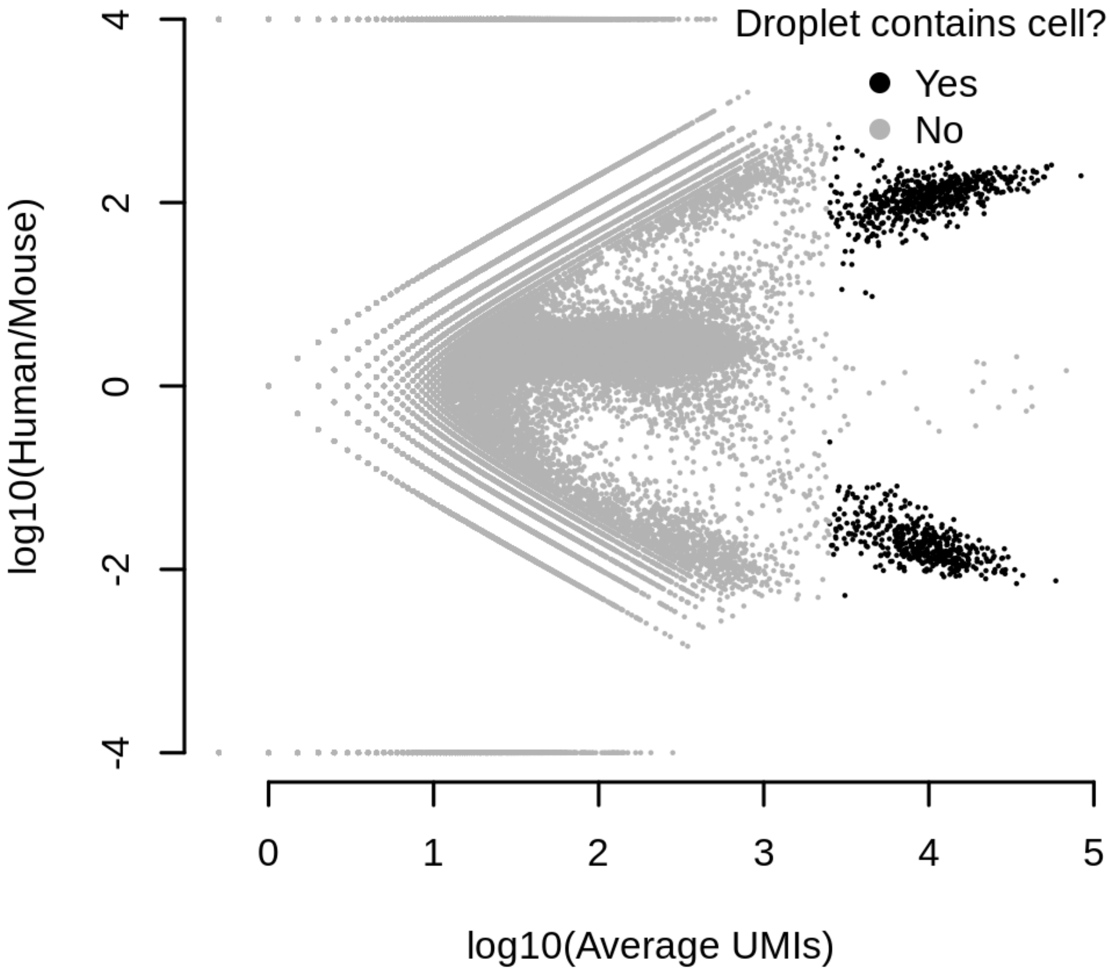

Supplementary Figure S3. The ratio of human to mouse transcripts on a log10 scale (y-axis) for all droplets in the DropSeq species-mixing experiment. Droplets containing cells are marked in black. The x-axis gives the average number of UMIs between human and mouse for each cell.
