# Supplementary material for: SoupX removes ambient RNA contamination from droplet-based single-cell RNA sequencing data
Source: Gigascience. 2020 Dec 26;9(12):giaa151. doi: 10.1093/gigascience/giaa151 (PMC7763177; doi:10.1093/gigascience/giaa151)
Supplement: giaa151_Supplemental_Figures_and_Tables [file giaa151_supplemental_figures_and_tables.zip › FigureS4.pdf]

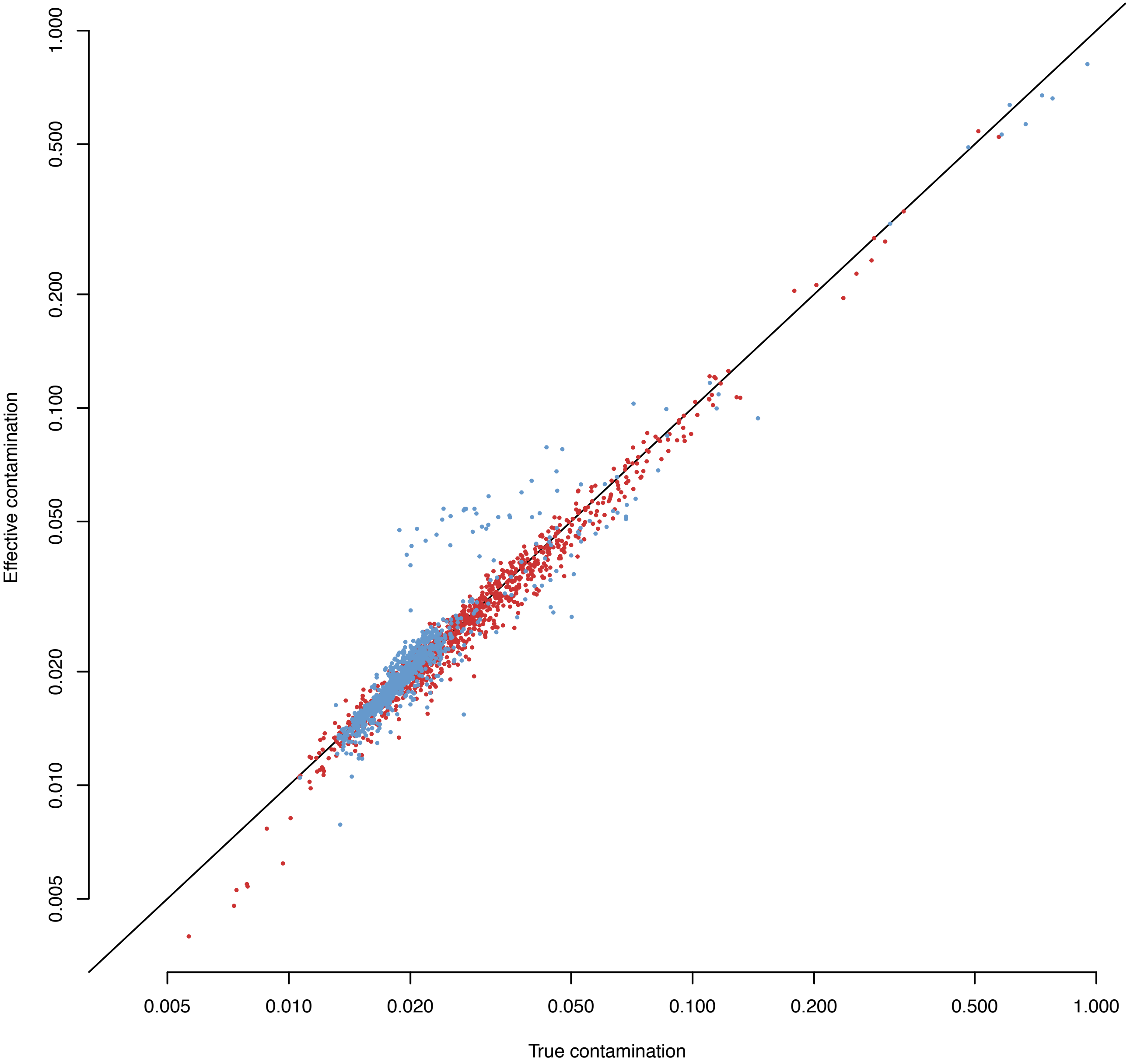

Supplementary Figure S4. The x-axis gives the true contamination rate measured using the cross-species transcripts in each cell. The y-axis gives the effective contamination rate obtained by applying SoupX at the cluster level using a constant global contamination rate, calculated as the fraction of removed counts by the application of SoupX. The line shows perfect correlation, and red and blue dots represent the 10X and DropSeq species-mixing experiments respectively.
