# Supplementary material for: SoupX removes ambient RNA contamination from droplet-based single-cell RNA sequencing data
Source: Gigascience. 2020 Dec 26;9(12):giaa151. doi: 10.1093/gigascience/giaa151 (PMC7763177; doi:10.1093/gigascience/giaa151)
Supplement: giaa151_Supplemental_Figures_and_Tables [file giaa151_supplemental_figures_and_tables.zip › FigureS5.pdf]

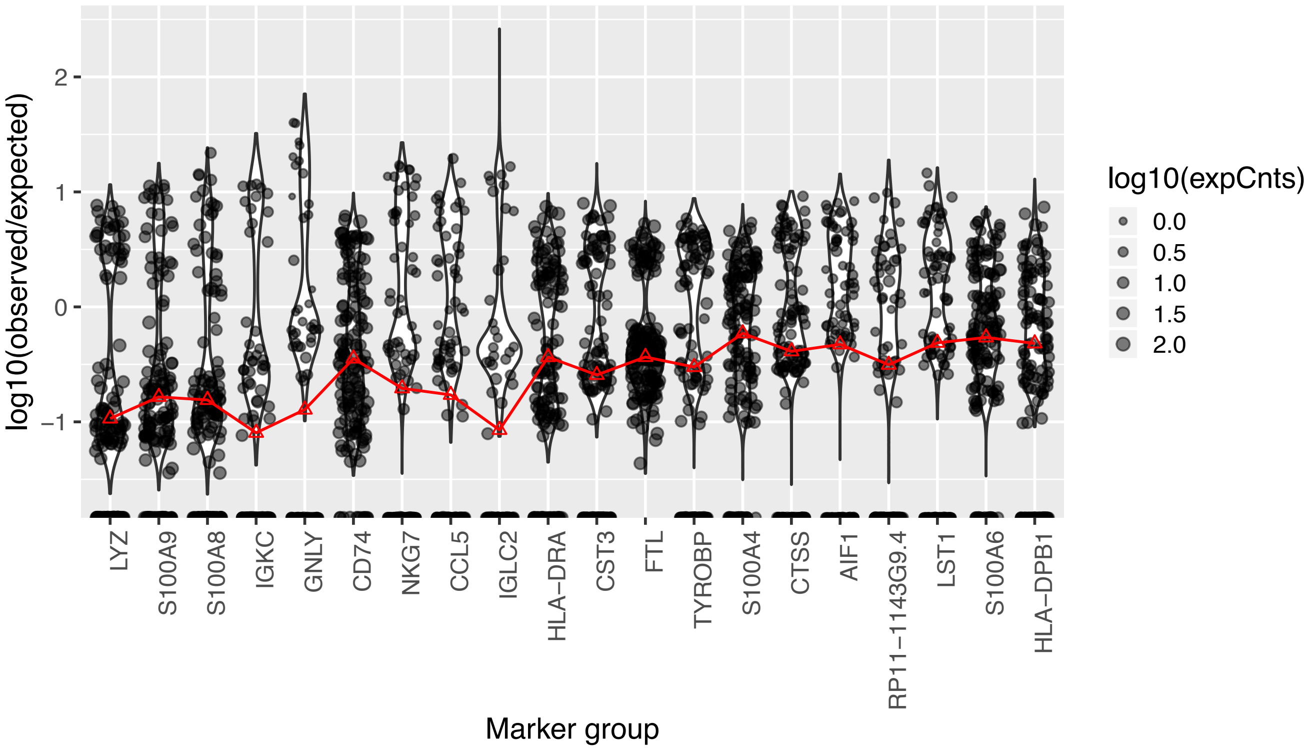

Supplementary Figure S5. Distribution of expression relative to background for genes in the PBMC data. The red line indicates the global estimate of the contamination fraction that would be obtained if just that gene were used to estimate contamination. Genes that are most useful for contamination estimation have a bimodal distribution, with cells genuinely expressing the gene yielding a value on the y-axis >0 and cells that do not express the gene having a value clustered around the true contamination rate.
