# Supplementary material for: SoupX removes ambient RNA contamination from droplet-based single-cell RNA sequencing data
Source: Gigascience. 2020 Dec 26;9(12):giaa151. doi: 10.1093/gigascience/giaa151 (PMC7763177; doi:10.1093/gigascience/giaa151)
Supplement: giaa151_Supplemental_Figures_and_Tables [file giaa151_supplemental_figures_and_tables.zip › FigureS6.pdf]

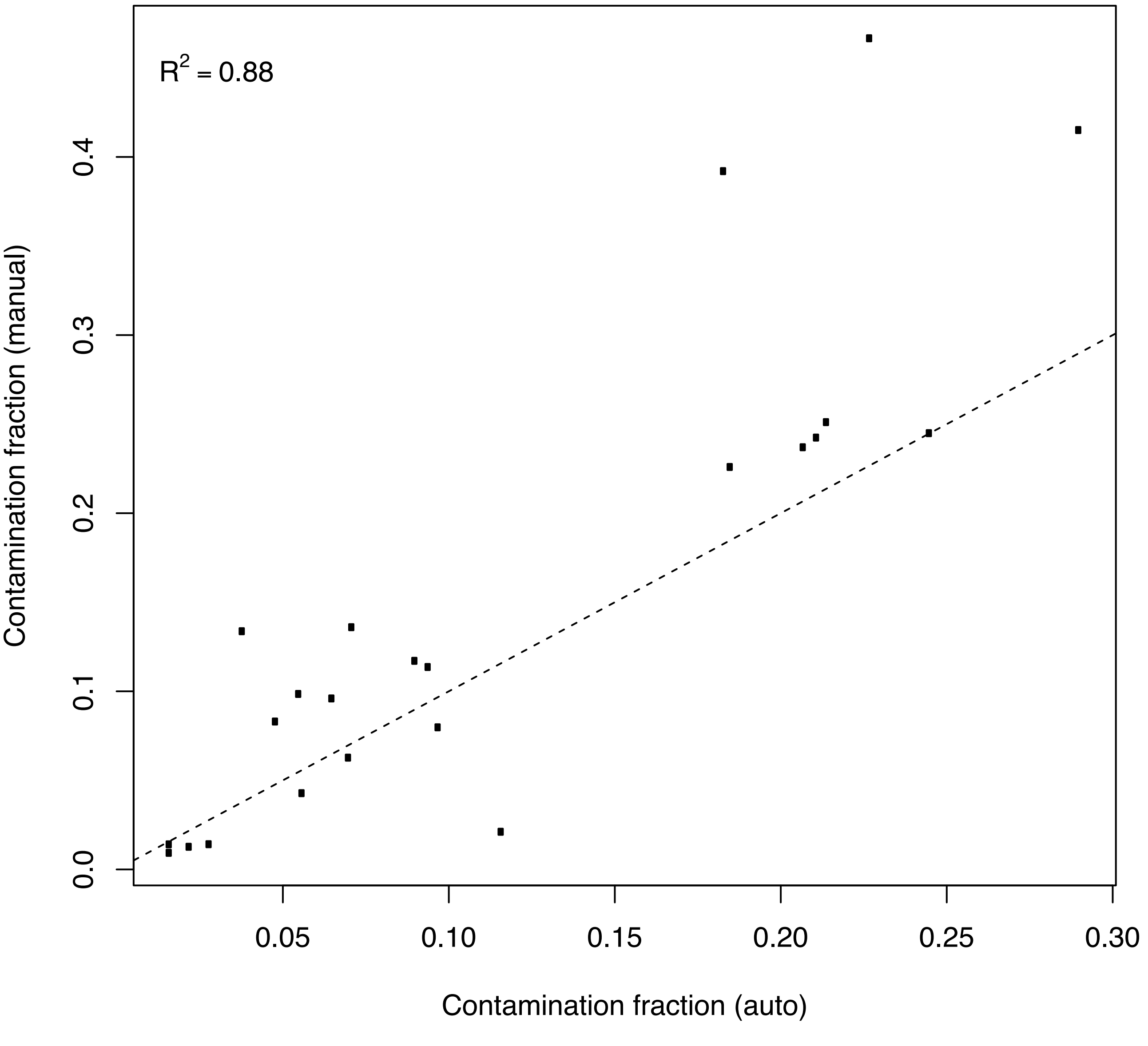

Supplementary Figure S6. Comparison of the contamination fraction estimated by the automated method (x-axis) and by manually supplying a gene set (y-axis), for each channel in the kidney tumour data. The dashed line indicates perfect correlation, and the Pearson correlation is shown in the upper left.
