# Supplementary material for: SoupX removes ambient RNA contamination from droplet-based single-cell RNA sequencing data
Source: Gigascience. 2020 Dec 26;9(12):giaa151. doi: 10.1093/gigascience/giaa151 (PMC7763177; doi:10.1093/gigascience/giaa151)
Supplement: giaa151_Supplemental_Figures_and_Tables [file giaa151_supplemental_figures_and_tables.zip › FigureS7.pdf]

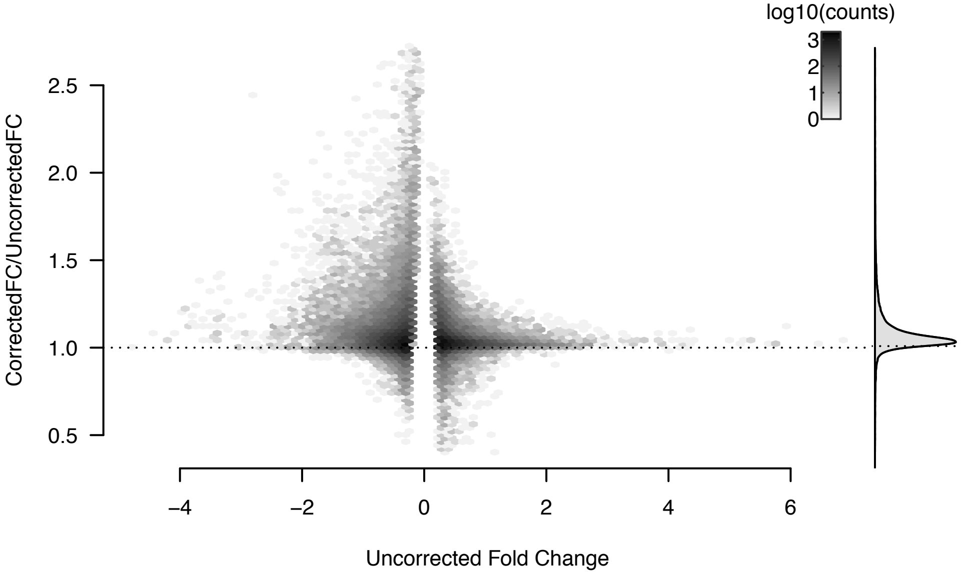

Supplementary Figure S7. The improvement in marker specificity following application of SoupX to the kidney tumour data. Note the different scale of the y-axis compared to Fig. 3. All genes that are markers of a cluster either before or after correction are identified, and their expression log fold change (FC) relative to the clusters that they do not mark is calculated before and after correction. The y-axis of this plot shows the fractional change in log FC after applying SoupX for all genes. Genes are grouped into bins for ease of representation, with the number of genes in each bin given by the colour scale. The marginal distribution across all genes is shown on the right, and the dotted line corresponds to no change in marker specificity after correction.
