# Supplementary material for: SoupX removes ambient RNA contamination from droplet-based single-cell RNA sequencing data
Source: Gigascience. 2020 Dec 26;9(12):giaa151. doi: 10.1093/gigascience/giaa151 (PMC7763177; doi:10.1093/gigascience/giaa151)
Supplement: giaa151_Supplemental_Figures_and_Tables [file giaa151_supplemental_figures_and_tables.zip › FigureS8.pdf]

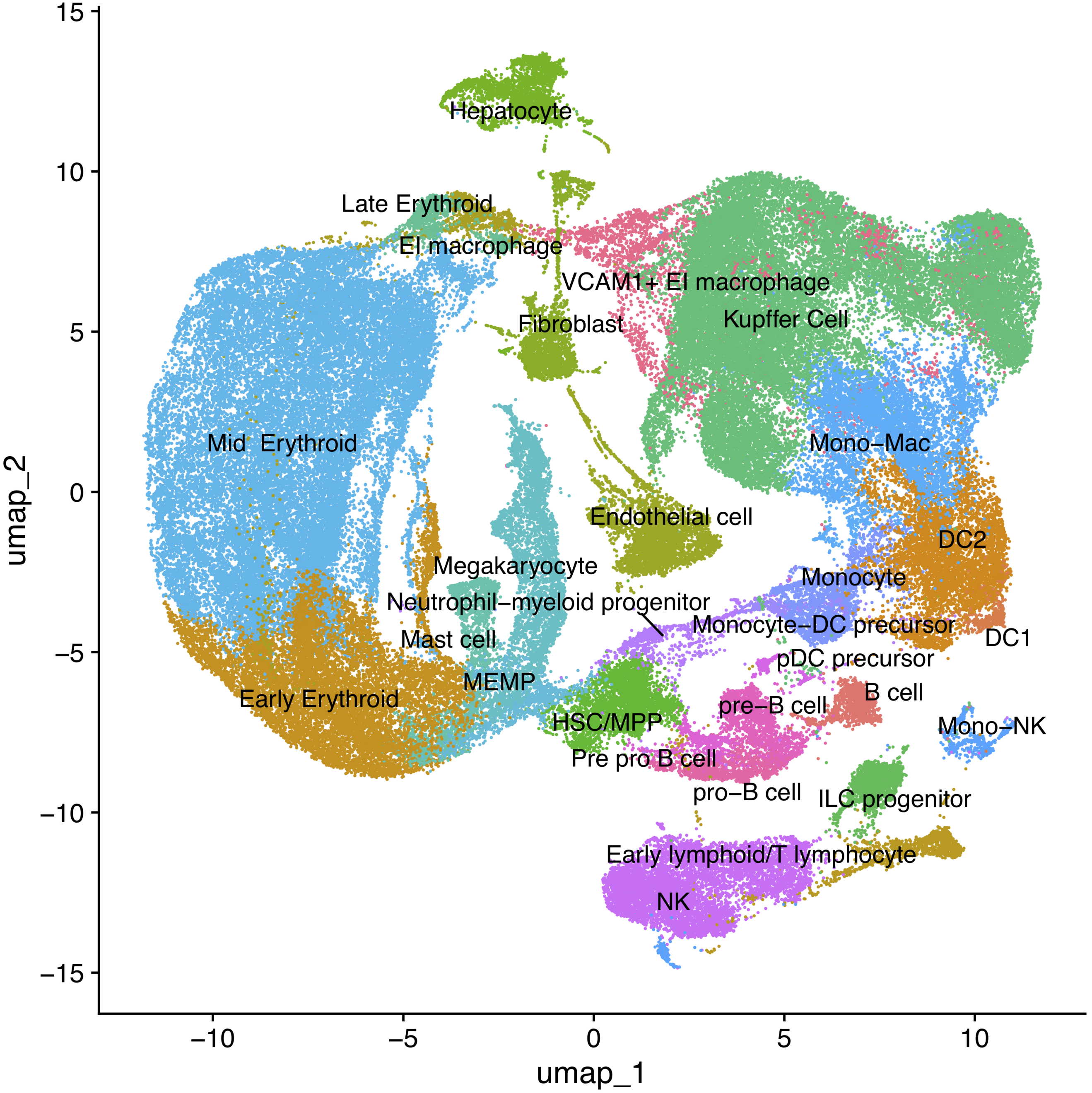

Supplementary Figure S8. Uniform manifold approximation and projection (UMAP) representation of the single-cell fetal data. Each point is coloured by its cell type and a cell type label is placed at the position of the average cell.
