# Supplementary material for: SoupX removes ambient RNA contamination from droplet-based single-cell RNA sequencing data
Source: Gigascience. 2020 Dec 26;9(12):giaa151. doi: 10.1093/gigascience/giaa151 (PMC7763177; doi:10.1093/gigascience/giaa151)
Supplement: giaa151_Supplemental_Figures_and_Tables [file giaa151_supplemental_figures_and_tables.zip › FigureS9.pdf]

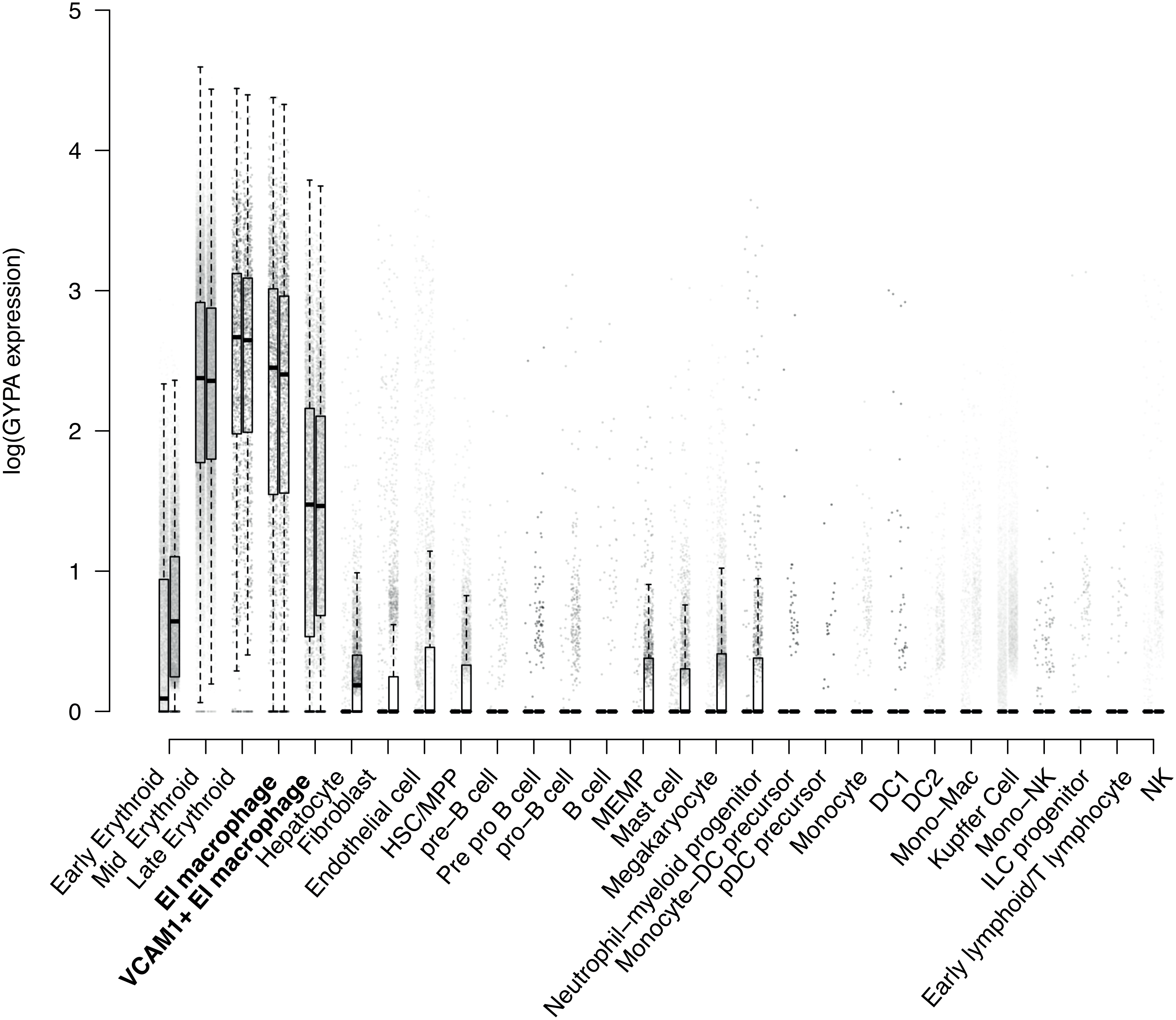

Supplementary Figure S9. Normalized gene expression of GYPA (y-axis) in fetal liver data by cell type before and after ambient RNA removal by SoupX (x-axis). The cell types on the x-axis represent the different cell types as annotation in Supplementary Fig. S8. For each cell type, box plots indicate the me- dian, quartiles, and 1.5 times the interquartile range for cells after SoupX correction (left) and before (right). For each distribution, each cell's expression is also shown with horizontal jitter and transparency inversely proportional to the number of cells of that type. The 2 EI Macrophage populations are emphasized in boldface.
