# Supplementary material for: SoupX removes ambient RNA contamination from droplet-based single-cell RNA sequencing data
Source: Gigascience. 2020 Dec 26;9(12):giaa151. doi: 10.1093/gigascience/giaa151 (PMC7763177; doi:10.1093/gigascience/giaa151)
Supplement: giaa151_Supplemental_Figures_and_Tables [file giaa151_supplemental_figures_and_tables.zip › giaa151-supp.pdf]

Supplementary Methods

Notation

We refer to the observed counts in a droplet c for gene g as n_{gc}. Sums taken over a variable are represented with a dot, so $n_{.c} = \sum_g n_{gc}$: the sum over all genes for droplet c. The quantity m_{gc} represents the cell endogenous counts present in a droplet c, for gene g. Similarly, o_{gc} represents the other counts in a droplet, contributed by background contamination.

We denote the fractional abundance of gene g in the soup or ambient mRNA background as b_g. This is defined such that $b_. = 1$. ρ_c represents the background contamination fraction in droplet c, defined as $o_{.c}/n_{.c}$.

We define $\mathcal{G}$ as the set of all genes that can be detected in a sequencing experiment.

Choice of count distribution

The most appropriate model for count-based data, such as the counts produced by scRNA-seq, is a multinomial distribution. This distribution provides the probability of observing a given partition of N counts into k genes, given the relative probabilities of each gene p_g.

In estimating how many counts to remove, where N tends to be small, we directly maximize the multinomial likelihood. However, in other cases we approximate the multinomial distribution as k Poisson distributions, which is an accurate approximation in the limit of large N and small p.

These distributions are commonly extended to include overdispersion by using a Dirichlet multinomial or negative binomial distribution. Throughout this article we ignore the effects of overdispersion for computational expediency and because for most estimation procedures used the maximum likelihood estimator does not depend on the overdispersion (e.g., estimating the mean).

Detailed description of the SoupX method

As discussed in the main text, SoupX aims to remove the contribution of the cell-free mRNA molecules from each cell. The algorithm consists of the following 3 steps:

(1) Estimate the ambient mRNA expression profile from empty droplets.
(2) Measure the contamination fraction, the fraction of UMIs originating from the background, in each cell.
(3) Correct the expression of each cell using the ambient mRNA expression profile and estimated contamination.

SoupX produces a modified table of counts, which can be used in place of the original count matrix in any downstream analysis tool. This supplement provides the details for each of these 3 parts of the SoupX method.

Background expression profiles

To calculate the expression profile of cell-free mRNAs, we assume that droplets with a very low UMI count contain only cell-free mRNAs. Because the number of droplets with low UMI counts is very large compared to the number of cells ($\sim 10^6$ droplets vs $\sim 10^4$ cells), there is typically abundant power to accurately calculate the expression profile of the cell-free background. Let $\mathcal{D}$ denote the set of all droplets with a UMI count $\alpha_l \leq n_{.c} \leq \alpha_u$. The background expression fraction for gene g, b_g, is estimated as

$$b_g = \frac{\sum_{c \in \mathcal{D}} n_{gc}}{\sum_{\tilde{g} \in \mathcal{G}} \sum_{c \in \mathcal{D}} n_{\tilde{g}c}}. \tag{S1}$$

That is, b_g is the fraction of counts derived from gene g in the set of empty droplets $\mathcal{D}$, normalized so that $b_. = 1$. In the species-mixing data, we could directly measure the background contamination in droplets *with* cells. We used this gold standard to measure the accuracy of the estimated background as a function of the number of UMIs in the droplets used to estimate it (Fig. S2). Based on this, we set $\alpha_l = 2$ and $\alpha_u = 10$ in this article. We ignore droplets with 1 UMI to prevent errors in the droplet barcodes from contaminating our estimate of the background (although we find no evidence that this is a problem for Chromium 10X data). Different cut-offs may be more appropriate for different technologies, but we find good correlation between all expression profiles generated using cut-offs up to $\sim$100 counts.

Calculating the contamination fraction

SoupX needs an estimate of the global contamination fraction present in a channel. This is generally not known in advance and must be estimated from the data or provided by the user. Our method approaches this problem by trying to identify a set of gene/cell pairs for which the cell endogenous expression can be assumed to be zero. That is, the task is to identify

$$\Omega = \{c, g | m_{gc} = 0\}. \tag{S2}$$

For genes and cells in Ω we see from Equation 2 that $n_{gc} = o_{gc}$; i.e., the observed counts are purely due to background contamination.

In certain circumstances (e.g., a well-annotated dataset with very specific marker genes) the set Ω may be able to be specified directly by the user. Where this is not the case, we construct Ω in a 2-step procedure.

First, a set of genes that are known to be very specific to a particular cell type and highly expressed in that cell type are identified. Typical examples of this are red blood cells and haemoglobin genes or IG genes and B cells. However, this set will depend on the experiment being performed; e.g., the insulin gene *INS* and pancreatic β cells will work well in the pancreas and be useless elsewhere.

Having identified a set of genes suitable for estimating the contamination fraction, we next identify which cells definitively do not express these genes. Again, the ideal way to do this is to have a well-annotated dataset where this decision can be specified in a biologically motivated way. For example, when using haemoglobin genes to estimate the contamination fraction, cells annotated as red blood cells can be excluded from Ω and other cell types included.

Where this is not possible we proceed by identifying all cells for which cell endogenous expression must be non-zero. These are the cells that are significant using a Poisson P-value under the null hypothesis that the $n_{.c}$ counts are distributed in the same way as in the background distribution. That is, we identify all cells where $P_c < 0.05$ and

$$P_c = \sum_{k=n_{gc}}^{\infty} \frac{\lambda^k e^{-\lambda}}{k!}, \tag{S3}$$

where $\lambda = \rho_{\max} b_g n_{.c}$ and $\rho_{\max}$ is the largest plausible contamination fraction (which is set to 1 by default). Put another way, this

identifies all cells for which the fraction of counts derived from the genes of interest in the cell exceeds the fraction of counts for those genes in the background contamination.

We then cluster the data and exclude any cluster that contains a cell for which $P_c < 0.05$. This conservative approach helps ensure that the cells used to estimate the contamination are those cells with zero endogenous expression of the target genes. This approach can be made more or less conservative by adjusting the P-value threshold or clustering more or less finely.

Having constructed Ω, the set of gene/cell pairs with which to calculate the contamination fraction, we calculate the global contamination fraction for an experiment as

$$\rho = \frac{\sum_{g,c\in\Omega} n_{gc}}{\sum_{g,c\in\Omega} n_{.c} b_g}. \tag{S4}$$

In most cases a global estimate of ρ is sufficient, and we find little evidence of large cell-to-cell variability in ρ. Furthermore, in most cases the counts available to estimate the contamination within each cell, $\sum_{g\,\in\,\Omega|c} n_{gc}$, is too low to provide an accurate cell-level estimate.

For cases where there is a need to estimate cell-specific contamination, we share information between cells using a hierarchical Bayes model. Under the model:

$$\mu \sim \text{Normal}(0, 0.5) \tag{S5}$$

$$\sigma \sim \text{Normal}(0, 1) \tag{S6}$$

$$\rho \sim \text{Normal}(-4, 1) \tag{S7}$$

$$\rho_c \sim \text{Normal}(\mu, \sigma) \tag{S8}$$

$$n_{gc} \sim \text{Poisson}(n_{.c} b_g(\rho + \rho_c)) \forall g, c \in \Omega. \tag{S9}$$

That is, the data are assumed to follow a Poisson distribution, with mean given by the expected background counts times a cell-specific contamination fraction. The cell-specific contamination fraction is modelled as a global contamination, plus some perturbation whose prior distribution is normally distributed, the parameters of which are determined from the data.

Automated estimation of the contamination fraction

In cases where the choice of gene set used to construct Ω and estimate ρ is not obvious, we provide an automated alternative. The intuition behind this approach is that the genes that are most often useful in estimating ρ are markers of cells in the dataset being investigated. In detail, we first cluster the data and identify highly specific marker genes of each cluster. To perform marker identification, we use an approach based on the term frequency, inverse document frequency (tf-idf) metric frequently used in natural language that has been successfully used previously [15].

Selecting marker genes for which tf-idf > 1 provides a list of genes that can be used to feed into the manual estimation procedure described above. This list is further refined by excluding any gene that is not expressed at or above the 99th expression quantile in the background profile b_g, as these genes provide the most accurate estimate of the contamination fraction. Each gene on this list is fed through the procedure above to identify Ω and estimate ρ, producing many quasi-independent estimates of ρ.

We then assume that inaccurate estimates of ρ will be randomly distributed, while true estimates will cluster around the true value. As such, the most common estimate of ρ amongst the set of estimates obtained from marker genes will represent the true value. Next we calculate the posterior probability of ρ given these estimates, utilizing a γ distribution prior with mean (SD) of 0.05 (0.1). This broad prior has little effect on the posterior distribution but can be set to be something more informative where prior information is available. Finally, ρ is set to the most likely value in the posterior distribution.

Correcting cell expression profiles

Having calculated the expression profile for the background b_g and the contamination fraction ρ, we use this information to modify the table of counts and remove contaminating mRNAs, m_{gc}. The obvious way to do this is by simply subtracting the contribution due to soup and setting $m_{gc} = n_{gc} - \rho n_{.c} b_g$ (or $m_{gc} = 0$ if $n_{gc} < \rho n_{.c} b_g$). Indeed, this is the maximum likelihood estimator of m_{gc} for Poisson-distributed counts with mean given by Equations 2 and 3.

However, following this approach will systematically undercorrect the data because the only counts for which the data will be modified are those for which $n_{gc} \geq \rho n_{.c} b_g$. To correct for this, more than $\rho n_{.c} b_g$ must be subtracted from those counts for which $n_{gc} > \rho n_{.c} b_g$. The reason for this is that the data must be modelled by a distribution that takes into account the competitive nature of sequencing, such as the multinomial distribution. That is, we need a statistical model that will find not just the most likely amount of contamination in each gene separately but will require that the total number of counts removed from each cell must equal $\rho n_{.c}$. The usual approach of modelling the counts for each gene/cell pair with a Poisson distribution approximates a multinomial distribution (similarly the often used negative-binomial distribution is an approximation of a Dirichlet-multinomial distribution). Therefore, the true problem that we want to solve is to maximize the multinomial likelihood of o_{gc} (the contaminating counts for gene g in cell c) with multinamial $n = o_{.c} = \rho n_{.c}$, and probabilities given by b_g, subject to the constraint that $0 \leq o_{gc} \leq n_{gc} \forall g$, c (i.e., we cannot remove more counts than we observe).

We solve this problem by recognizing that gene expression "buckets" are filled in an order depending on their expression and their expression in the background. That is, as the number of counts removed increases, counts will be completely removed from genes in an order determined by n_{gc}/b_g. Knowing this, it is straightforward to calculate which genes will be completely removed for a given number of total contaminating counts and then distribute the remaining counts to be removed between all other genes proportionally to b_g.

This procedure is followed independently for each cell to produce modified counts. Where integer counts are required for downstream analysis, we round the corrected counts up to the nearest integer with probability given by $m_{gc} - \lceil m_{gc} \rceil$.

Improving count removal using clustering

Where clustering of cells is provided, the above procedure can be improved by applying the correction procedure to counts aggregated within clusters. Doing this greatly increases the statistical power to distinguish between contamination and true expression. The value of ρ used for cluster P is calculated as

$$\rho_P = \frac{\sum_{c\in P} n_{.c}\rho_c}{\sum_{c\in P} n_{.c}} \tag{S10}$$

and the number of contaminating counts for each gene is calculated as above.

To redistribute the calculated contaminating counts to the single-cell level, counts for gene g are distributed to each cell

with weights given by $n_{.c}\rho_c$. This redistribution is done using the same logic as for removing counts to ensure that a cell cannot be assigned more contamination for a gene than has actually been observed.

Cells that have a higher true contamination rate than the global average will have more non-zero counts in genes with high contamination than cells with a lower contamination rate than average. Because of this, the redistribution procedure described above will assign more contaminating counts to high-contamination cells and fewer to low-contamination cells, even without this information being explicitly provided. This can be seen in Fig. S4, where the effective cell-level contamination rate implied by correcting at the cluster level and redistributing counts is highly correlated with the true cell-level contamination rate.

Processing of datasets

The DropSeq Species Mixing experiment was downloaded from the SRA (SRR1748411) and quantified using Alevin[1] with a mixed human/mouse reference and the "forceCells" flag set to 1 to include all barcodes.

For all 10X datasets, we used all droplets identified by cellranger as containing cells. In the species-mixing data, we removed any droplet with ≥1,000 UMIs from *both* human and mouse genes because these are likely doublets. For the DropSeq species-mixing experiment we set this threshold to 5,000.

We used the Seurat package (http://satijalab.org/seurat/) to parse distinct cell types and marker genes from these preprocessed sequencing data. Raw counts of UMIs per gene in each cell were normalized using the Seurat::NormalizeData function, to implement the transformation

$$x_{gc} = \log(1 + 10^4 \hat{f}_{gc}), \qquad \text{(S11)}$$

where $\hat{f}_{gc}$ is the observed proportion of UMIs in droplet c from gene g; x_{gc} is the library-size–normalized expression of gene g in droplet c.

Variable genes were identified using the Seurat::FindVariableGenes function with default parameters. Within Seurat, we subset the library-size–normalized gene expression matrix on the variable genes, and we standardized the matrix so that the variable genes have mean (SD) of 0 (1). We calculated the first 30 principal components of the standardized matrix, and the graph-based clustering algorithm implemented in Seurat::FindClusters evaluated the distance between cells in this 30D principal component analysis volume. The tSNE embedding was calculated using a perplexity of 30, and clusters were identified with the Seurat::FindClusters resolution parameter set to 1.

To identify genes specific to each cluster, we used the Seurat "FindMarkers" function with default parameters.

These markers were then manually inspected and each cluster was assigned a cell type based on the comparison of these markers to the literature (particularly [15, 2, 3, 4]).

For the fetal liver data, we used pre-supplied UMAP coordinates and cell labels, generated as previously described [16]. Contamination fractions were determined independently for each channel using the automated method, except for those channels where this could not return a result owing to too few cells, in which case we manually set the contamination to 10%, which was roughly the average of all other channels.

References

1. Srivastava A, Malik L, Smith T, et al. Alevin efficiently estimates accurate gene abundances from dscRNA-seq data. Genome Biol 2019;20(1):65.

2. Chabardès-Garonne D, Mejéan A, Aude JC, et al. A panoramic view of gene expression in the human kidney. Proc Natl Acad Sci U S A 2003;100(23):13710–5.

3. Habuka M, Fagerberg L, Hallström BM, et al. The kidney transcriptome and proteome defined by transcriptomics and antibody-based profiling. PLoS One 2014;9(12):e116125.

4. Lee JW, Chou CL, Knepper MA. Deep sequencing in microdissected renal tubules identifies nephron segment-specific transcriptomes. J Am Soc Nephrol 2015;26(11):2669–77.
