# Supplementary material for: SoupX removes ambient RNA contamination from droplet-based single-cell RNA sequencing data
Source: Gigascience. 2020 Dec 26;9(12):giaa151. doi: 10.1093/gigascience/giaa151 (PMC7763177; doi:10.1093/gigascience/giaa151)
Supplement: giaa151_Response_to_Reviewer_Comments_Original_Submission [file giaa151_response_to_reviewer_comments_original_submission.pdf]

Author's Response To Reviewer Comments

Close

Dear Dr. Hans Zauner,

Please find attached our revised manuscript. As previously discussed, in this revision we have included additional data derived from a different biological context (human fetal liver) to further demonstrate the biological utility of SoupX. To ensure that this demonstration of utility is well motivated, we focus on issues raised during peer review of the fetal liver paper, which SoupX is able to definitively resolve. Also as requested, we have included a discussion of how SoupX relates to other ambient mRNA correction tools that have been published since SoupX was first written.

We provide a point by point response to the points raised by the original reviewers and Dr. Varodom Charoensawan in the attached supplementary document. Although we do not agree with all the points raised by reviewers, the process has undoubtedly improved the manuscript. Thank you for your patience with the further COVID associated delay and we hope that you agree that our revised manuscript adequately addresses the remaining concerns with our manuscript.

With best wishes,

Matthew Young and Sam Behjati

Close
