# Supplementary material for: SoupX removes ambient RNA contamination from droplet-based single-cell RNA sequencing data
Source: Gigascience. 2020 Dec 26;9(12):giaa151. doi: 10.1093/gigascience/giaa151 (PMC7763177; doi:10.1093/gigascience/giaa151)
Supplement: giaa151_Response_to_Reviewer_Comments_Revision_1 [file giaa151_response_to_reviewer_comments_revision_1.pdf]

Author's Response To Reviewer Comments

Close

A formatted, point by point response to reviewer comments are provided in the document marked as "Supplementary Material" entitled Response to reviewers, round 3".

Close
