# Supplementary material for: SoupX removes ambient RNA contamination from droplet-based single-cell RNA sequencing data
Source: Gigascience. 2020 Dec 26;9(12):giaa151. doi: 10.1093/gigascience/giaa151 (PMC7763177; doi:10.1093/gigascience/giaa151)
Supplement: giaa151_Reviewer_2_Report_Original_Submission — Chris L Plaisier, Ph.D -- 2/21/2020 Reviewed [file giaa151_reviewer_2_report_original_submission.pdf]

Reviewer Report

Title: SoupX removes ambient RNA contamination from droplet-based single-cell RNA sequencing data

Version: Original Submission **Date:** 2/21/2020

Reviewer name: Chris L Plaisier, Ph.D

Reviewer Comments to Author:

The ideas represented in SoupX are interesting and would likely be of interest to the general single cell community if they could address a couple of major issues in the current tool implementation. The reformatting of the methods section and inclusion of more useful formulas with more thorough descriptions is very helpful. However, the authors have chosen to take out of the paper the selection of genes to be used in calculating the background. The previous heuristic based approach was criticized as it difficult to assess whether the complicated heuristic approach they employed would be generalizable or if there would be cases where it might fail. The decision to remove this part from the SoupX tool makes it incomplete, and detracts significantly from its usability.
Major Critiques:
1. There needs to be statistical assessments to demonstrate the benefit of using SoupX on a dataset. Currently there is only anecdotal evidence given to suggest the method works as intended. These statistical tests are vital in determining the utility of the SoupX tool.
2. The lack of appropriate datasets to test the tool are something the authors must address. If there really are no datasets which would help them to test their tool then how can we ever know the tool actually functions appropriately? The authors should devise experiments that would allow them to do so and conduct them so they can test their tool.
3. Can the authors make the heuristic for selecting genes more simple or demonstrate at least that the complexity is necessary by applying it to datasets and proving its performance improvement over more simplistic approaches.
4. Is the approach generalizable? Please prove it with statistical assessments.

Level of Interest

Please indicate how interesting you found the manuscript: Choose an item.

Quality of Written English

Please indicate the quality of language in the manuscript: Choose an item.

Declaration of Competing Interests

Please complete a declaration of competing interests, considering the following questions:

- Have you in the past five years received reimbursements, fees, funding, or salary from an organisation that may in any way gain or lose financially from the publication of this manuscript, either now or in the future?
- Do you hold any stocks or shares in an organisation that may in any way gain or lose financially from the publication of this manuscript, either now or in the future?
- Do you hold or are you currently applying for any patents relating to the content of the manuscript?
- Have you received reimbursements, fees, funding, or salary from an organization that holds or has applied for patents relating to the content of the manuscript?
- Do you have any other financial competing interests?
- Do you have any non-financial competing interests in relation to this paper?

If you can answer no to all of the above, write 'I declare that I have no competing interests' below. If your reply is yes to any, please give details below.

I declare that I have no competing interests

I agree to the open peer review policy of the journal. I understand that my name will be included on my report to the authors and, if the manuscript is accepted for publication, my named report including any attachments I upload will be posted on the website along with the authors' responses. I agree for my report to be made available under an Open Access Creative Commons CC-BY license (http://creativecommons.org/licenses/by/4.0/). I understand that any comments which I do not wish to be included in my named report can be included as confidential comments to the editors, which will not be published.

Choose an item.

To further support our reviewers, we have joined with Publons, where you can gain additional credit to further highlight your hard work (see: https://publons.com/journal/530/gigascience). On publication of this paper, your review will be automatically added to Publons, you can then choose whether or not to claim your Publons credit. I understand this statement.

Yes Choose an item.
